# Low-Molecular-Weight Compounds Produced by the Intestinal Microbiota and Cardiovascular Disease

**DOI:** 10.3390/ijms251910397

**Published:** 2024-09-27

**Authors:** Lorena Cuervo, Patrick L. McAlpine, Carlos Olano, Javier Fernández, Felipe Lombó

**Affiliations:** 1 Research Group BIOMIC (Biosynthesis of Antitumor Molecules), Departamento de Biología Funcional, Área de Microbiología, Universidad de Oviedo, 33006 Oviedo, Spain; lorenacuervodelpozo@gmail.com (L.C.); olanocarlos@uniovi.es (C.O.); 2 IUOPA (Instituto Universitario de Oncología del Principado de Asturias), 33006 Oviedo, Spain; 3 ISPA (Instituto de Investigación Sanitaria del Principado de Asturias), 33006 Oviedo, Spain; 4 Research Group BIONUC (Biotechnology of Nutraceuticals and Bioactive Compounds), Departamento de Biología Funcional, Área de Microbiología, Universidad de Oviedo, 33006 Oviedo, Spain

**Keywords:** short-chain fatty acid, hydrogen sulfide, trimethylamine N-oxide, phenylacetylglutamine, bile acids

## Abstract

Cardiovascular disease is the main cause of mortality in industrialized countries, with over 500 million people affected worldwide. In this work, the roles of low-molecular-weight metabolites originating from the gut microbiome, such as short-chain fatty acids, hydrogen sulfide, trimethylamine, phenylacetic acid, secondary bile acids, indoles, different gases, neurotransmitters, vitamins, and complex lipids, are discussed in relation to their CVD-promoting or preventing activities. Molecules of mixed microbial and human hepatic origin, such as trimethylamine N-oxide and phenylacetylglutamine, are also presented. Finally, dietary agents with cardioprotective effects, such as probiotics, prebiotics, mono- and poly-unsaturated fatty acids, carotenoids, and polyphenols, are also discussed. A special emphasis is given to their gut microbiota-modulating properties.

## 1. Introduction

Cardiovascular diseases (CVDs) are one of the leading causes of death, encompassing various conditions such as ischemic heart disease, ischemic stroke, intracerebral hemorrhage, and others.

The prevalence of CVDs varies significantly by region, but it is estimated that approximately 523 million people worldwide suffer from CVDs. There is an annual incidence of 17.9 million new cases and about 18.6 million deaths, with 85% of these deaths attributable to heart attacks and strokes, constituting 32% of all deaths globally. About 75% of deaths due to CVDs occur in low- and middle-income countries, whereas high-income countries have seen a decline in CVD mortality rates in recent years due to improvements in healthcare systems and preventive measures [1,2].

Moreover, significant differences are observed in age and gender. There is a marked increase in the prevalence of CVDs in individuals over 70 years of age. Men are at a higher risk of developing CVDs compared to women, and within the female population, the risk escalates in postmenopausal women. This is due to the loss of estrogen and its residual cardioprotective effect by preventing the dysregulation of the β-adrenergic receptors expressed in the heart [3,4].

The primary risk factors for CVD are mainly certain chronic diseases such as hypertension, hyperlipidemia, diabetes mellitus, and obesity; as well as lifestyle-related factors such as physical inactivity, high alcohol consumption, and smoking; or environmental factors, such as ambient air pollution [5,6]. Among these, the most significant risk factor is hypercholesterolemia, characterized by elevated levels of cholesterol in the blood, particularly low-density lipoprotein cholesterol (LDL-C). Elevated levels of these cholesterol molecules in the blood lead to their penetration into the endothelial lining of arteries, resulting in their oxidation. Oxidized LDL (oxLDL) is then phagocyted by macrophages, transforming them into foam cells and leading to the formation of fatty streaks and arterial stenosis. Over time, the accumulation of these fatty streaks results in the development of atherosclerotic plaques, which consist of a necrotic lipid core and a fibrous cap. At this stage, the arterial walls harden and lose elasticity. When an atherosclerotic plaque ruptures, it can lead to the occlusion of the vessel lumen, forming a thrombus and potentially causing events such as myocardial infarction or ischemic stroke [7,8,9].

Gut microbiota dysbiosis is able to trigger these oxidative stress effects, therefore, contributing to the development of atherosclerosis. Several genera have been described as more abundant in the gut microbiota of CVD patients, such as the bacterial phyla *Proteobacteria*, and *Fusobacteria*, or the genera *Gardnerella* (*G. vaginalis*), *Acidaminococcus* (*A. fermentans*), *Pseudomonas* (*P. aeruginosa*), *Necropsobacter* (*N. massiliensis*), *Prevotella* (*P. copri*), *Veillonella*, *Streptococcus*, *Desulfovibrio*, *Moraxella*, and *Actinomyces* [10,11,12], as well as the fungi *Fusarium* and *Issatchenkia* [13].

**Figure 1:**
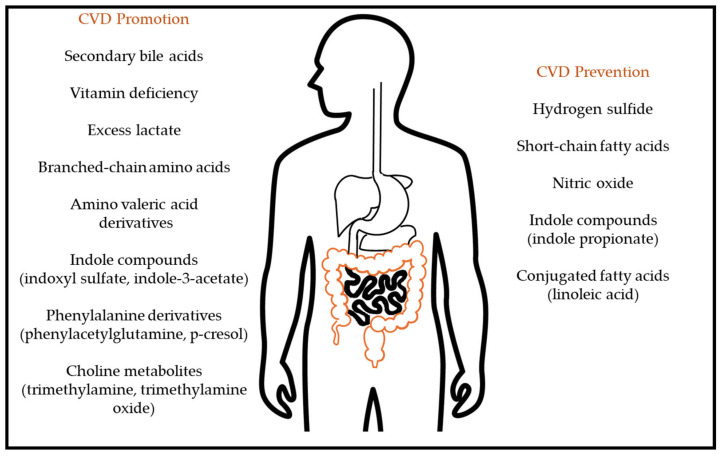
Main metabolites that cause promotion (column “CVD Promotion”) and prevention (column “CVD Prevention”) of cardiovascular events.

Some of the biochemical and metabolic reasons linking this gut dysbiosis with CVD are known thanks to in vivo experiments. For example, using germ-free mice with a defined known gut microbiota lacking the *cutC* gene, which is involved in the conversion of dietary choline into trimethylamine (see Section 3.1), the addition of a *Clostridium sporogenes* strain expressing *cutC* was enough to trigger the formation of this compound (and also of its hepatic oxidized derivative). This further enhanced the rate of thrombus formation in these animals [14]. The gut microbiota can influence CVD in different ways, including the modulation of inflammation and vascular function through lipopolysaccharides, the promotion of atherosclerosis, thrombosis, and fibrosis through trimethylamine-oxidized products and phenylacetylglutamine, changes in blood pressure and myocardial repair through short-chain fatty acids, fibrosis, and damages to renal function through indoles and modulation of lipid and glucose metabolism through bile acids [14]. The gut mucosa barrier function can be altered under certain conditions, due to improper function of the tight junctions between epithelial cells, giving rise to a leaky gut status, and its association with pro-inflammatory conditions. This pathology also shows a reduction in the mucus layer that protects and separates the intestinal epithelium from the microorganisms in the lumen and contains abundant antimicrobial peptides and immunoglobulin A [15]. The final effect of these alterations is the translocation of bacterial cells and their components (such as lipopolysaccharides) from the gut lumen to the submucosa, therefore, activating the immune system toll-like receptors. This happens especially under dysbiotic conditions, such as an overgrowth of *Proteobacteria* taxa [14,15]. Some bacterial taxons contribute to enhancing this gut barrier function, such as *Faecalibacterium prausnitzii*, *Lactobacillus* spp. which increase the expression of occludin and zonulins, *Escherichia coli* Nissle 1917 which enhances the expression of zonulins, and *Bifidobacterium* spp. which stabilize claudins and occludin [15]. Also, bacterial metabolites such as butyrate enhance the function of tight junctions [15]. The major molecules discussed in this review are summarized in Figure 1.

In this review, a detailed analysis of the current knowledge on metabolites of influence on cardiovascular disease, via its promotion or its prevention, has been carried out, using as reference database PubMed (https://pubmed.ncbi.nlm.nih.gov/, accessed on 19 March 2024). The search topics were the following ones: cardiovascular disease, microbiota, prevention, promotion, short-chain fatty acid, conjugated fatty acid, indole, nitric oxide, hydrogen sulfide, bile acid, branched-chain amino acid, choline metabolite, and phenylalanine metabolite.

## 2. Low-Molecular-Weight Compounds and Cardiovascular Disease Prevention

### 2.1. Short-Chain Fatty Acids

Short-chain fatty acids (SCFAs) are molecules that consist of two to six carbon atoms in a single chain ending in a carboxyl group. The three most abundant SCFAs in the human gut are acetate, butyrate, and propionate [16], accounting for over 90% of the SCFAs content in human feces and existing at a ratio of 3:1:1, respectively [17]. They are present in numerous dietary sources [18] but are best known for being fermented by the intestinal microbiota in the presence of non-digestible prebiotic fiber [19,20,21,22]. They play roles in numerous aspects of human health, even accounting for approximately 10% of human caloric requirements [16,23], and are one of the best-studied classes of microbiome-derived metabolites. The interaction of SCFAs with host tissues and their impact on host physiology has been extensively studied. Thus, numerous reviews have already conducted in-depth analyses of the impacts of SCFAs on different aspects of CVD [24,25,26,27,28,29,30,31,32,33]. For this reason, and due to the broad scope of this work, this section on SCFAs will only focus on presenting the most recent discoveries linking SCFAs with dyslipidemia, atherosclerosis, type II diabetes, hepatic malignancies, and hypertension.

#### 2.1.1. Acetate

Acetate is the most abundant SCFA in the human gut. It is also the simplest SCFA, consisting of 2 C atoms. It is produced by a wide range of gut microbes and achieves relatively high concentrations in blood (130–200 μM) [34,35,36]. About two-thirds of gut acetate is generated by prebiotic fiber fermentation (using pyruvate and then acetyl-CoA as earlier precursors) in the colon and one-third via bacterial acetogenesis (acetate formation from formic acid or carbon dioxide plus hydrogen) [35]. Its impact on cardiovascular health has been studied in the contexts of dyslipidemia, atherosclerosis, type II diabetes, liver disease, and hypertension.

Studies in murine models have demonstrated that dietary acetate supplementation has positive effects. In models of obesity, acetate supplementation has been observed to decrease blood triglyceride and total cholesterol concentrations while increasing glucagon-like peptide-1 (GLP-1) and leptin concentrations [37]. GLP-1 is a hormone that is secreted by intestinal enteroendocrine cells in response to glucose and stimulates insulin secretion [38], while leptin is secreted by adipocytes and induces feelings of satiety. Thus, indicating that this supplementation leads to a better response to dietary glucose consumption and less hunger. Furthermore, acetate supplementation has also been found to reduce kidney damage, fibrosis, and inflammation in type II diabetes models [39], as well as blood pressure and heart rate in hypertension models [40]. When supplemented in a mixture with propionate and butyrate, it was observed to reduce liver damage in a model of non-alcoholic steatohepatitis (NASH) as measured by decreased circulating levels of the transaminases aspartate aminotransferase (AST) and alanine aminotransferase (ALT), as well as decreased hepatic macrophage infiltration [41]. These effects seem to be mediated by G protein-coupled receptor 43 (GPR43), indicating its role as an acetate receptor. GPR43 activation has been associated with increased glucose uptake, upregulation of glycogen synthesis genes, and increased expression of glucose transporters in the HepG2 hepatic cell line. Other mechanisms include inhibiting inflammatory responses in cell lines, modulating sympathetic tone, and increasing expression of fatty acid oxidation genes in hepatic tissue [42].

In contrast to the positive results in murine models, human clinical findings have been much more mixed, although still largely positive. While increases in blood acetate concentrations due to dietary interventions have been associated with decreased total cholesterol and low-density lipoprotein (LDL), as well as increased high-density lipoprotein (HDL) [43,44], blood acetate levels in type II diabetic adults have been found to correlate directly with the number of common femoral artery plaques [45]. Thus, associations between blood acetate levels and disease markers need to be analyzed in the context of the disease model and population in which they are measured. For example, in healthy adults, it has been demonstrated that blood acetate concentrations correlate inversely with blood glucose and insulin concentrations [46], while in type II diabetic adults, blood acetate concentrations correlate directly with blood glucose concentrations [45]. Studies in hypertensive patients have found that blood acetate concentrations are higher in treatment-resistant subjects than in treatment-responsive subjects [47], while fecal acetate concentrations are higher in hypertensive patients than in normotensive controls [48,49].

#### 2.1.2. Propionate

Although it is not as abundant as acetate, propionate is still one of the three main SCFA in terms of abundance in the human body. It is slightly larger than acetate, with 3 C atoms. This SCFA is mainly produced in the gut by genera such as *Bacteroides*, *Prevotella*, *Alistipes*, *Roseburia*, *Eubacterium*, *Blautia*, *Coprococcus*, *Dialister*, *Phascolarctobacterium*, and *Akkermansia* [50]. Murine models of propionate treatment have been very promising. Studies have shown that oral propionate treatment can reduce blood LDL levels and reduce dietary cholesterol uptake by limiting the expression of the known cholesterol transporter NPC1 (Niemann-Pick disease, type C1) in colon cells [51]. It even has demonstrated anti-hypertensive properties such as reduced arterial pressure, cardiac hypertrophy, fibrosis, and vascular dysfunction, in addition to anti-atherosclerotic properties via an immune modulating mechanism where T cells and macrophages are decreased in brachiocephalic artery sections. Surprisingly, all of these effects are not observed in T-cell-depleted mice, indicating that immune system modulation is likely one of the critical mechanisms by which propionate’s cardioprotective effects occur [52]. In addition to these studies in murine models, one mechanistic study in the HepG2 liver cell line found that propionate treatment decreased glucose production and prevented insulin signaling, although this was not associated with an increase in cellular glycogen stores [53].

As in the case with acetate, results in human models are much more mixed than what is seen in the murine models. Results have indicated that blood propionate concentrations are significantly lower in patients with coronary artery disease than in healthy controls [54], and these concentrations correlate inversely with body mass index [55]. Similarly, hypercholesterolemic adults who were prescribed dietary fiber supplementation experienced reduced blood LDL levels, and those participants who did not respond to the fiber treatment had significantly less abundance of SCFA-producing genera in their fecal microbiotas. Furthermore, fecal propionate levels post-treatment correlated inversely with blood LDL levels [56]. In contrast, other dietary modification studies in hypercholesterolemic adults have yielded conflicting results. In one study, blood propionate concentrations correlated inversely with blood cholesterol and LDL [43], while in a second study, they correlated directly with LDL and inversely with HDL [44]. More conflicting results indicate that low fecal propionate levels are associated with a greater risk of developing type II diabetes [57], while blood propionate concentrations correlate with increased blood leptin, LDL, and blood pressure [46]. Even in blood pressure studies, results are very conflicting. Some studies have found that blood propionate levels correlate with higher blood pressure [46,58], while others have observed that serum propionate is lower in hypertensive patients than in normotensive controls [47].

In a rather damning set of studies, female mice supplemented with sodium propionate experienced increased blood glucose and reduced hepatic glycogen stores. The authors associated this finding with elevated activation of the sympathetic nervous system via increased blood norepinephrine levels. These results were further confirmed in humans by giving participants a meal containing sodium propionate and found that it resulted in increased serum norepinephrine and glucagon [59].

#### 2.1.3. Butyrate

Butyrate is the largest of the big three SCFAs found in the human body, consisting of 4 C atoms. It is mainly produced in the gut by genera such as *Eubacterium*, *Roseburia*, *Anaerostipes*, *Coprococcus*, *Faecalibacterium*, and *Subdoligranulum* [50] While it is not as abundant as acetate, it may be the most studied compound in this group in terms of CVD. Murine models of butyrate supplementation have found that it successfully reduces blood cholesterol [60], body weight, body fat [61], fasting blood glucose concentrations [62], aortic atherosclerotic plaque area [60], hepatic steatosis [60,61], fibrosis, collagen content, blood ALT levels [63], blood pressure [40], cardiac hypertrophy, and fibrosis [64]. It has also been shown to increase aortic relaxation [65], hepatic glycogen stores [42,62], as well as cardiac angiogenesis [66], and contractile function [67].

Mechanistically, butyrate appears to have a remarkably diverse repertoire of effects on the body. It appears to have immune-modulating activities, such as stimulating macrophages to increase cholesterol efflux into HDLs [60]. It also directly alters adipose tissue activity by reducing endoplasmic reticulum stress, T-cell invasion [68], and cytokine expression [69], as well as acting as an energy source for adipocytes, where it increases thermogenesis, mitochondrial respiration, and conversion to brown fat [70,71]. Furthermore, it has been shown to increase glucose uptake in adipose tissue due to increased expression of the Glut-2 transporter [72]. In the liver, it has been observed to increase apolipoprotein ApoA1 gene expression [73], although other studies have found that this did not lead to actual ApoA1 secretion into culture media [74]. ApoA1 is one of the main components of HDL, so greater ApoA1 biosynthesis could help to increase circulating HDL concentrations. Butyrate treatment has also been shown to reduce hepatic uric acid concentrations as well as the expression of pro-inflammatory genes in hepatic cells [69,75]. As it was seen previously with acetate, many of these hepato-protective effects are not seen in the absence of GPR43 (also known as FFAR2, free fatty acid receptor 2) or GPR41 (also known as FFAR3, free fatty acid receptor 3), indicating the importance of both these receptors for SCFA signaling in general [62,76]. In a study of HUVEC endothelial cells that were co-cultured with THP-1 monocytes, it was found that sodium butyrate treatment reduced oxidative stress, production of cytokines, as well as VCAM-1 and E-selectin gene expressions. VCAM-1 and E-selectin are known cell adhesion proteins, and their reduced expression resulted in reduced monocyte adhesion to the endothelial cells, which is a crucial part of atherosclerotic plaque formation [77]. In cardiac tissue, sodium butyrate supplementation has been seen to increase ATP reserves, indicating its potential function as an energy source for cardiac tissue [67].

In human trials, butyrate has shown interesting effects in relation to exercise. Sodium butyrate supplementation has been found to augment the effects of physical exercise in obese adolescents [78], while supplementation alone has been found to induce similar effects to exercise alone [66]. The potential link between exercise and butyrate is GPR43. Studies in diabetic mice have shown that the blood glucose-lowering effects of exercise are inhibited when GPR43 is inhibited [79], just as it has been observed in numerous SCFA supplementation studies that have already been discussed.

In further studies, blood butyrate concentrations have been found to correlate inversely with body mass index, while total blood SCFA concentrations correlate inversely with blood glycerol, free fatty acids, and triacylglycerol concentrations [55]. In addition, sodium butyrate supplementation in diabetic patients has been found to reduce inflammatory markers in circulating macrophages [80], while in normoglycemic adults, the butyrate production potential of their gut microbiota has been found to associate with better insulin responses to an oral glucose tolerance test [57].

In contrast to all these positive results, a large number of negative results must be discussed as well. In murine models, results may be highly sex-specific. Studies in rats have found that males experience reduced blood cholesterol with sodium butyrate treatment, while females not only do not experience this benefit [81] but also experience increased serum fatty acids and triglycerides [82]. These negative results have also been observed in numerous human studies. A study found that sodium butyrate supplementation did not reduce serum cholesterol or triglycerides in diabetic adults [83]. At the same time, a dietary modification study that found that blood acetate concentrations correlated inversely with LDL and positively with HDL also found that blood butyrate levels had the opposite correlation [44]. In terms of atherosclerotic activity, a study of adults with type II diabetes and peripheral arterial disease positively correlated blood butyrate concentrations with the number of popliteal artery plaques [45], while a study in vascular smooth muscle cells found that treatment with sodium butyrate increased cellular calcification while sodium acetate and sodium propionate treatments had no effect [84]. In terms of hypertension, blood butyrate concentrations were higher in hypertensive patients than in normotensive controls, in contrast to blood propionate concentrations, which were lower in hypertensive patients than in normotensive controls [47]. Relatedly, some studies have found that fecal acetate, butyrate, and propionate levels are higher in hypertensive patients than in normotensive controls [48,49].

#### 2.1.4. Other Short-Chain Fatty Acids

As previously stated, acetate, butyrate, and propionate make up over 90% of the SCFAs in the human body. In the 10% that remains, other low-abundance SCFAs have been less studied. Valerate (a 5 C atom SCFA), for example, consists of a butyl group bound to a carboxyl group. In hypercholesterolemic patients, blood valerate concentrations have been found to correlate inversely with HDL [44], as well as correlate directly with the number of superficial femoral artery plaques, popliteal artery plaques, and blood glucose concentrations in diabetic patients [45]. In contrast, hexanoate (6 C atoms SCFA) has been found to correlate directly with HDL and inversely with LDL in hypercholesterolemic patients [44].

#### 2.1.5. Short-Chain Fatty Acids in Context

SCFAs are canonically considered to be good, health-promoting compounds, but the large number of negative associations that have been described in the previous sections bring this view into question. It is important to consider SCFAs in the context of the gut microbiome and intestinal physiology. First, it is fundamental to differentiate oral SCFA consumption from that derived from the gut microbiome. Orally consumed SCFAs are almost certainly absorbed in the small intestine [85], whereas microbially synthesized SCFAs are produced in the colon. This difference may have major implications for the physiological action of these compounds. It is well demonstrated that butyrate functions as the primary energy source for colon epithelial cells [86,87,88]. Furthermore, it has been demonstrated in vitro that the effects of butyrate upon ApoA1 gene expression in hepatic HepG2 cells are significantly attenuated when the butyrate must first pass through caco-2 colon cells [89]. In addition, studies in vivo have observed that butyrate concentrations are highest in the proximal colon and are much lower in feces and even blood [19,90]. Furthermore, the role of the vagus nerve must be considered. It has been observed that intracolonic sodium butyrate injections induce a greater reduction in blood pressure than intravenous injections, but that the effects of the intracolonic injection are prevented by severing the vagus nerve [91]. Thus, it seems likely that the impact of butyrate upon human physiology occurs primarily due to its interaction with cells in the colon and the vagus nerve. Oral butyrate supplementation bypasses these processes entirely and may lead to entirely different physiological effects by interacting with other organs and nerves.

Secondly, it is important to consider the interaction between SCFAs and the gut microbiome itself. Acetate has been described as a major modulator of gut microbiome metabolism [92]. Studies have shown that the majority of butyrate-producing bacteria preferentially utilize acetate produced by other species for growth [93]. Meanwhile, acetate production in the gut microbiome has been shown to alter concentrations of other gut microbiota metabolites that are involved in CVD such as trimethylamine oxide and p-cresol (discussed later in this review) [94]. Thus, butyrate biosynthesis may actively reduce acetate concentrations, while high acetate concentrations may increase the abundance of butyrate-producing bacteria and increase the butyrate production potential that is observed in metagenomic studies. Thus, the biosynthesis potential of the various SCFAs should not be considered in isolation, but rather in the context of each other.

Finally, several studies have demonstrated that fecal SCFA concentrations do not necessarily correlate with their blood concentrations, and some have even indicated that fecal concentrations correlate inversely with blood concentrations [55,94]. Furthermore, studies have indicated that fecal SCFA concentrations may not correlate with metabolic parameters [55]. If it is assumed that intestinal SCFAs are rapidly absorbed [90,95], then it is possible to consider that the concentration of SCFAs in feces is not only a measure of microbial biosynthesis but also of intestinal absorption. It is entirely possible that high SCFA concentrations in feces are due to a decreased capacity for their uptake in certain individuals, which could lead to less beneficial effects. Furthermore, if it is assumed that the majority of bodily SCFAs are present in colon tissue, with relatively low amounts in circulation (as stated in a previous paragraph), it is possible that high blood concentrations of SCFAs are not necessarily due to greater microbial biosynthesis. A less efficient utilization of these SCFAs in colon tissue, or potentially greater intestinal permeability, could lead to higher blood SCFA concentrations. These last two statements are purely hypothetical and require further research, but they could help to explain some of the conflicting results that have been discussed previously. 

### 2.2. Hydrogen Sulfide (H_2_S)

Hydrogen sulfide (H_2_S) and its derivatives have crucial systemic-level implications as activators of diverse cellular cascade pathways, regulators of post-translational modifications, and modulators of redox states. In addition, its involvement in cardiovascular protection has recently aroused great interest, given its anti-inflammatory, antioxidant, pro-angiogenic, antiapoptotic, vasodilating, and cardioprotective properties [96]. It is a major component of the sulfur cycle in the environment and is essential for living beings, since it is involved in the biosynthesis of amino acids, proteins, and enzymes [97], which means that despite its toxicity in higher concentrations, it is biosynthesized by the body in micromolar concentrations. Due to this toxicity, H_2_S is a double-edged sword, with its catabolism being extremely important to counterbalance its biosynthesis, to maintain the correct physiological concentration. Sulfur can occur in different forms in animals, due to its wide range of oxidation states, but H_2_S is the main form of interest because of its cardiovascular involvement, acting as a reductant in its gaseous form and diffusing freely under acidic conditions, such as those found in ischemic pathology. Nevertheless, its ability to react with other oxidized or reduced groups is determined by whether it is in this neutral state or in a charged anionic state, which is its major physiological form [98]. This metabolite has a normal concentration in the human body of 0.1 to 0.8 μM and its half-life varies from a few seconds to minutes [99]. H_2_S, nitric oxide (NO), and carbon monoxide (CO) make up the three molecules known as gastrotransmitters, which trigger responses in various organ systems by activating complex cascade pathways [96]. In particular, H_2_S interacts with NO, increasing the signaling activity as a neurotransmitter since they have similar and interrelated functions in the cardiovascular system, as well as the tangent signaling pathways [98].

Not only does H_2_S have beneficial systemic effects, but other sulfur-derived compounds such as cysteine persulfide (CysSSH) and glutathione persulfide (GSSH) have been described as potent antioxidants acting against reactive oxygen or nitrogen species (RONS) and free radicals [100], participating thereby in relevant signaling functions in physiological and pathophysiological conditions. Several pathways can produce different metabolites derived from H_2_S [98]. Nevertheless, it is known that between 20% and 25% of the sulfur-containing organic compounds that are ingested through diet are metabolized to H_2_S [96]. It is estimated that the human colon harbors between 0.17 and 0.38 mmol/L of H_2_S [101].

Endogenous H_2_S can be produced by both enzymatic and non-enzymatic pathways, and its biosynthesis is mostly determined by the enzymes cystathione γ-lyase (CTH) and cystathione β-synthase (CBS) and the sequential activity of cysteine aminotransferase (CAT) and 3-mercaptopyruvate sulfurtransferase (MPST), using cysteine and its derivatives as substrates [101]. Cysteinyl tRNA synthetase is another enzyme that can produce hydropersulfides (RSSH) and polysulfides (RS_n_R). In addition, sulfite reductase [NADPH] flavoprotein α-component (CysJ) and anaerobic sulfite reductase subunit A (AsrA) can reduce oxidation products back to H_2_S [98].

Nevertheless, there is a certain amount of H_2_S produced by other routes such as the activity of intestinal bacterial microbiota, which can produce H_2_S using cysteine as a sulfur source or via dissimilatory reduction of inorganic substrates [97]. Sulfate-reducing bacteria (SRB) are anaerobic microorganisms that make up a part of normal gut microbiota. They constitute a highly variable group in morphological and nutritional terms, but in common, they require inorganic sulfate as an electron acceptor to obtain energy from organic compounds [97,102]. The aforementioned reduction of sulfate to produce H_2_S, using H_2_ as an electron donor [101], and a microbial imbalance towards this type of bacteria, is related to numerous inflammatory diseases [103]. These microorganisms mainly use the oxidized state of inorganic sulfur from complex organic substrates such as those obtained from food [102]. *Desulfovibrio*, *Desulfobacter*, *Desulfobulbus*, and *Desulfotomaculum* are genera involved in reducing sulfur using sulfate as a substrate. Via the ATP sulfurylase, ATP and sulfate form adenosine 5′-phosphosulfate, which is then reduced to AMP and sulfite by the APS reductase pathway. *Desulfovibrio piger* is the most frequent species of SRB present in the human colon [101], while *Campylobacter* and *Bilophila* are related to the use of sulfite as a substrate [97]. Given its high permeability across the intestinal membrane, H_2_S produced by the gut microbiota can have a very significant influence on general physiology, both for beneficial and detrimental effects [104]. The excess of this compound can turn favorable effects into disadvantageous ones due to genotoxic effects, causing inhibition of mitochondrial respiration in colonic cells and promoting carcinogenesis by acting as a growth factor [96]. Despite its toxic nature, there are numerous pleiotropic implications in H_2_S at the systemic level depending on its concentration, such as promoting or relieving inflammatory processes. Endogenous production of H_2_S is highly regulated, and it has even been described that a deficiency in some of the enzymes involved in H_2_S production, such as due to certain polymorphisms, can be compensated by the others. This is why, even though this compound is endogenously produced by epithelial cells, production by the microbiota can turn favorable effects into pathological processes since the generation of this compound via microbial metabolism of dietary sulfur-derived substrates is higher than that emerging from the endogenous metabolism alone [104,105].

#### 2.2.1. Cysteine Metabolism

Dietary proteins, either intact or partially digested in the small intestine, are transferred to the colon [100,103], where they act as a source of amino acids, including cysteine. The amino acids are then used by the gut microbiota to give rise to different metabolites, including H_2_S. In this way, dietary consumption has a direct impact on the final concentration of H_2_S in the body and determines its production by the microbiota. Most amino acids are absorbed by the colonic mucosa, as well as different derivatives, which can be transformed, and subsequently released into the bloodstream, where they can exert an effect on other distant parts of the body. Various genera such as *Fusobacterium*, *Clostridium*, *Escherichia*, *Salmonella*, *Klebsiella*, *Streptococcus*, *Desulfovibrio*, and *Enterobacter* are able to metabolize cysteine into H_2_S with the enzyme cysteine desulfhydrase [103].

#### 2.2.2. Clinical Implications in Cardiovascular Disease

H_2_S has been described as having a protective role in the cardiovascular system since basal amounts of this compound are necessary for health maintenance and little changes can trigger inflammatory processes, which are considered biomarkers of CVD risk [98]. Modulation of H_2_S, or exogenous incorporation of it, enhances cardiac function and promotes angiogenesis, arteriogenesis, vasodilation, and blood flow in ischemic limbs [96]. Additionally, it inhibits platelet aggregation, cardiomyocyte apoptosis [98], and the onset of atherosclerosis and cardiac hypertrophy, as well as reducing the production of reactive oxygen species (ROS) due to its antioxidant properties. Patients with coronary disease have shown particularly low concentrations of this molecule, and treatments with H_2_S have been reported to reduce pathological effects. In the case of ischemic reperfusion injury, exogenous infusion of H_2_S reduced infarct size and preserved ventricular function [98].

In vivo studies in animal models have demonstrated that exogenous H_2_S treatment is effective in treating myocardial hypertrophy [106], myocardial ischemia and reperfusion [107], and heart failure [108]. Some of the identified mechanisms of protection include interactions with other molecules such as NO, heme, and antioxidant molecules. Others include protein post-translational modification by sulfhydration [109] and induction of signaling cascades [98]. Furthermore, regulation of H_2_S concentrations through dietary modulation has been demonstrated due to the existence of compounds that are capable of binding H_2_S and reducing its free concentration, as well as dietary patterns, such as caloric restriction, which regulate the expression of genes involved in H_2_S biosynthesis, such as CBS [110].

## 3. Low-Molecular-Weight Compounds and Cardiovascular Disease Promotion

### 3.1. Trimethylamine N-Oxide (TMAO)

Trimethylamine N-oxide, also known as TMAO, is one of the most studied microbiota-associated metabolites in association with CVD promotion. At the time of writing, a PubMed search of TMAO and CVD returned almost 1000 publications since 2019 alone. Detailed descriptions of the TMAO function are available in other review articles devoted to the topic [111,112,113,114,115,116,117,118,119,120,121,122,123,124,125,126,127,128,129,130,131,132]. Here we will discuss the most recent findings associating TMAO production from the gut microbiota and its implications in CVD.

**Figure 2:**
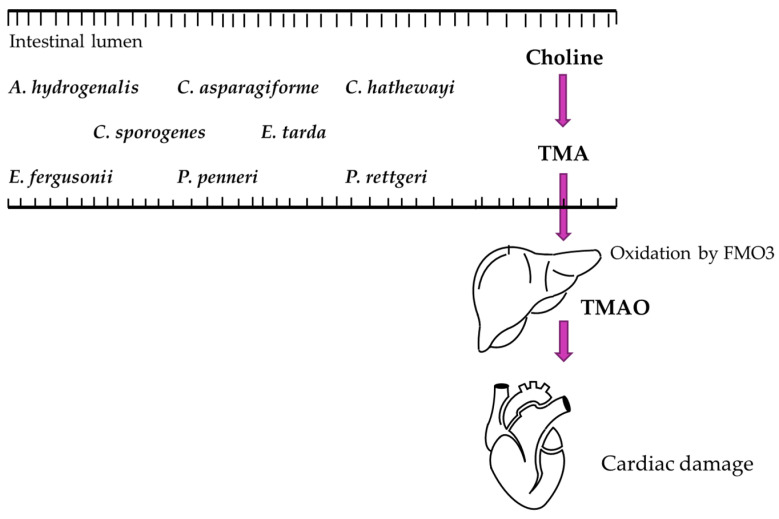
Overview of TMAO biosynthesis.

TMAO is produced when a liver enzyme converts trimethylamine (TMA) into TMAO. TMA is a product of microbial metabolism of dietary choline, betaine, and carnitine; molecules that are primarily found in meat and eggs [133]. At least eight bacterial species have been identified to produce TMA in the human gut including *Anaerococcus hydrogenalis*, *Clostridium asparagiforme*, *Clostridium hathewayi*, *Clostridium sporogenes*, *Edwardsiella tarda*, *Escherichia fergusonii*, *Proteus penneri*, and *Providencia rettgeri* [134]. Once produced, TMA enters circulation and is transported to the liver via the portal vein. In the liver, it is oxidized to TMAO by flavin-containing monooxygenase 3 (FMO3) [135]. Once formed, TMAO circulates through the body until it is eliminated via glomerular filtration [136]. The TMAO biosynthesis pathway is summarized in Figure 2.

Diverse nutraceuticals, such as polyphenols, are able to reduce plasma TMAO levels, most probably due to their inhibitory effect on gut populations of some *Clostridium* taxons involved in TMA biosynthesis from dietary precursors [137]. Some of these compounds include resveratrol and flavonoids (catequins, anthocyanidin polymers, etc.), such as those present in grape products and extracts, which have been demonstrated to reduce TMAO serum concentrations (up to 63.6% reduction) in healthy persons after oral administration of 300 mg grape pomace extract [138].

Recent studies in clinical cohorts have associated blood TMAO concentrations with all causes of death [139,140], adverse cardiovascular events [141,142], heart failure [141,143,144], atherosclerosis, and related complications such as arterial stiffness and calcification [145,146,147,148,149,150,151,152], aortic aneurysm, stroke [153,154,155,156], type II diabetes [157,158,159], dyslipidemia, metabolic syndrome [160,161], and hypertension [162,163,164]. Furthermore, meta-analyses have confirmed the associations between blood TMAO levels and cardiovascular mortality [165], adverse cardiovascular events, heart failure [166], stroke [167], hypertension and diabetes [168], and higher risk of major adverse cardiovascular events (re-infarction, heart failure, mortality) in patients suffering myocardial infarction and showing, after hospitalization, plasma TMAO levels higher than 3.45 μM [169].

In addition, a great deal of effort has gone into elucidating the mechanisms by which TMAO induces these adverse cardiovascular pathologies. Numerous studies in murine models have investigated the impact of TMAO by dietary supplementation, gavage, and intravenous injection. In these studies, TMAO treatment was shown to reduce aortic endothelial permeability [170]; increase atherosclerotic plaque area, serum lipids, aortic root inflammation [171], arterial cell wall thickness [172], blood cytokines and triglycerides; reduce HDL [173]; induce abdominal aortic aneurysm and degradation of elastin [174]; and finally increase serum insulin levels and alter adipose tissue metabolic activity [175]. In addition, in one study, mice were treated to reduce their brown adipose tissue content, and they experienced significantly elevated blood TMAO levels and cardiac dysfunction [176], indicating a role for this type of tissue in TMAO homeostasis.

Research has also focused on understanding the impact of consumption of TMAO precursors such as choline and TMA. Studies in murine models with dietary choline supplementation have found elevated blood TMAO levels and similar physiological impacts to TMAO supplementation [171]. It has been observed that choline supplementation increases blood TMAO levels and reduces proline/serine-rich coiled-coil protein 1 (PSCR1) gene expression in peripheral blood mononuclear cells (PBMCs). PSCR1 knockout mice go on to experience increased atherosclerotic plaque area, and it has been demonstrated that PSCR1 is necessary for PBMC cholesterol transport. Thus, one of TMAOs potential mechanisms is to reduce PSCR1 expression and, thus, cholesterol transport into PBMCs and out of circulation [145]. Further confirmation of the pro-atherogenic properties of dietary choline has been found by the observation that adding dietary charcoal supplements to mouse diets reduces the bioavailability of dietary choline and reduces much of the associated cardiac dysfunction [177]. Finally, TMA supplementation in murine models has been found to induce elevated blood TMAO levels and significantly increased blood pressure readings [178].

In vitro studies have further confirmed these results in cellular and tissue models. Aortic valve interstitial cells treated with TMAO have been seen to experience endoplasmic reticulum and mitochondrial stress [152]. In contrast, bovine aortic endothelial cells treated with TMAO did not experience mitochondrial stress but did exhibit a reduced purinergic response, which is critical for vasodilation [179]. Furthermore, vascular smooth muscle cells treated with TMAO experienced elevated VCAM-1 gene expression levels, which have been known to increase macrophage adhesion [180], as well as a phenotypic transition that is associated with atherosclerosis development [181]. Finally, treatment of fatty liver cells with TMAO increased lipid deposition and expression of fibrosis-related genes [182]. Of great significance is the finding that HUVEC endothelial cells treated with TMAO experience greater FMO3 gene expression [172]. Since this enzyme is responsible for the conversion of TMA to TMAO, TMAO levels may induce a feedback loop where TMAO stimulates the conversion of TMA to more TMAO, and so on. It should be noted that the conversion of TMA to TMAO normally occurs in the liver, so the impact of FMO3 expression in non-hepatic tissue needs further investigation. In addition to these findings in cellular models, the treatment of human umbilical artery rings with TMAO and TMA has been shown to induce ring contraction [183]. Thus, the mechanisms by which TMAO may cause CVD appear to be very diverse, affecting a variety of different cell types and tissues.

Although most of the data indicate that TMAO is a promoter of CVD, some recent studies have found conflicting results. One genome-wide association study (GWAS) conducted in healthy adults found multiple polymorphisms that are associated with elevated TMAO levels. Strangely though, these polymorphisms did not correlate with the incidence of CVD [184]. Further studies have found that blood TMAO levels in patients do not significantly correlate with adverse cardiovascular events [185], endothelial function, cardiovascular risk factors [186], or cardiovascular death [187]. Furthermore, obese women undergoing an exercise regimen and dietary restriction have been found to experience decreased blood TMAO levels due to the treatment, but only in women who had significantly elevated levels to begin with [188]. Contrary to what would be expected is the fact that blood TMAO levels normally increase after gastric bypass surgery, and this increase is regarded as being positive. This association has been confirmed by finding that the increase in blood TMAO concentrations after surgery in obese patients is associated with a healthier blood lipid profile [189].

An additional paradox in this story is the impact of fish consumption. Fish meat is known to contain high levels of TMAO, and its consumption has been demonstrated to cause a transient rise in blood TMAO levels, which would logically associate fish consumption with an elevated risk of CVD [190]. In sharp contrast to this expectation, multiple studies have found the exact opposite result, with one associating fish consumption with elevated blood TMAO levels but not with the risk of adverse cardiovascular events [142]. This has been further corroborated by the comparison of Japanese participants from high seafood-eating and low seafood-eating regions of Japan, with the finding that participants from the high seafood-eating region had higher blood TMAO levels and less atherosclerotic plaque area and carotid intima–media thickness [191]. The authors also noted higher blood levels of the omega-3 fatty acids eicosapentaenoic acid (EPA) and docosahexaenoic acid (DHA). While the impact of omega-3 fatty acids will be discussed in greater detail in a later section, they are generally regarded as beneficial for CVD prevention, so this could be a potential mechanism by which fish consumption counteracts the effects of elevated TMAO.

In addition to all of this, questions remain about the health impacts of dietary choline consumption. While numerous studies discussed previously have associated choline consumption with increased TMAO levels and increased risk of CVD, some recent studies have brought this into doubt. One study in mice found that dietary choline consumption did increase blood TMAO levels but did not correlate with any markers of heart disease [192], while a second study in mice found that choline consumption actively reduced hypertension [193]. Furthermore, a separate study in mice found that dietary choline supplementation had similar fat mass-reducing effects to dietary butyrate supplementation while atherosclerotic plaques were unaffected [194]. Finally, eggs are known to be high in choline and cholesterol, but their impact on cardiovascular health is hotly debated. One small study in young patients found that egg consumption did not increase blood TMAO levels and improved vascular function [195].

While the exact impacts of choline, TMA, and TMAO may be under some debate, the pathway by which TMA and TMAO are produced is well understood and known to be heavily influenced by each individual’s specific microbiota. It has been demonstrated that red meat consumption only affects blood TMAO levels in participants who have a large abundance of TMA-producing bacteria in their intestinal microbiotas [196]. Furthermore, it has also been found that the abundance of a microbial gene cluster responsible for the conversion of γ-butyrobetaine (γBB) to TMA directly correlates with blood TMAO levels [197]. More functionally, it has been observed that *Lachnoclostridium saccharolyticum* is a bacterium that correlates with elevated TMAO levels in human subjects and that treating mice with it, along with dietary choline, induces significantly elevated blood TMAO levels and aortic plaque buildup compared to controls that only receive choline supplementation [198]. Furthermore, mice with induced periodontitis have been seen to experience elevated blood TMAO and liver FMO3 levels, as well as an increased number of atherosclerotic plaques [199], indicating potential involvement of the oral microbiota in CVD onset. In addition to the impact of the microbiota, studies in heart failure patients have found that certain polymorphisms of the FMO3 gene are significantly associated with an increased risk of cardiovascular death [144]. Thus, a large portion of the heterogeneity in the results of dietary choline intake upon heart disease may well be mediated by variation in the participant’s gut microbiotas and individual genetic differences.

Seeing the potential impact of the microbiota–FMO3 pathway in CVD progression, treatments targeting it to prevent TMAO synthesis have shown some promise. In murine models, numerous studies have investigated the use of 3,3-dimethyl-1-butanol (DMB), an inhibitor of the trimethylamine lyase enzyme, to prevent the conversion of choline to TMA. This treatment has been demonstrated to reduce blood TMAO levels and pulmonary hypertension [164], increase aortic diameter [154], reduce aortic valve thickness [152], increase cardiac ejection fraction [200], reduce atrial inflammation and susceptibility to atrial fibrillation [201], and reduce general blood pressure and norepinephrine levels [202]. In long-term treatments with DMB on adult mice (18 months old), this compound has shown a reduction in vascular parameters associated with aging and cardiovascular disease in older mice (27 months), such as aortic stiffness, NO production reduction, and increased superoxide production [203]. Furthermore, the use of broad-spectrum antibiotics to eliminate the host’s microbiota has been demonstrated to be as effective as DMB treatment [154].

While the exact impact of TMAO on cardiovascular health needs further research, the large number of studies associating it with negative health outcomes indicate its importance as a potential marker of cardiovascular health. Still, due to genetic and microbial variation between individuals, what may be bad for some may have no impact on others. Thus, the importance of personalized medicine may play center stage here. A deeper understanding of how the microbiota is associated with TMAO pathology will be necessary to allow for individualized microbiota-based interventions, which could be key to preventing TMAO-associated CVD.

### 3.2. Phenylacetylglutamine (PAG)

Beginning in 2018, numerous researchers using non-targeted metabolomics strategies began to identify phenylacetylglutamine (PAG) as a potential marker of CVD. It was found that blood PAG levels served as a marker for obesity [204] as well as for abnormally elevated risk of atherosclerotic plaque area [205]. In addition, it was also found to be a urine marker for type II diabetes [206]. Then, in 2020, a wide-ranging study by Nemet et al. demonstrated that circulating PAG levels served as a marker of adverse cardiovascular events including myocardial infarction, stroke, and death. Furthermore, they identified a mechanism of action where PAG acts as an agonist of the adrenergic receptors α2a, α2b, and β2 on blood platelet cells. Both in cellular and murine models, this was found to induce adhesion and aggregation. In addition, microbiota depletion in mice reduced circulating PAG concentrations, indicating its status as a microbiota-derived molecule. Finally, the microbial gene *porA* from *Clostridium sporogenes*, which forms phenylacetic acid from phenylpyruvic acid, was found to be necessary for PAG production in vivo [207].

**Figure 3:**
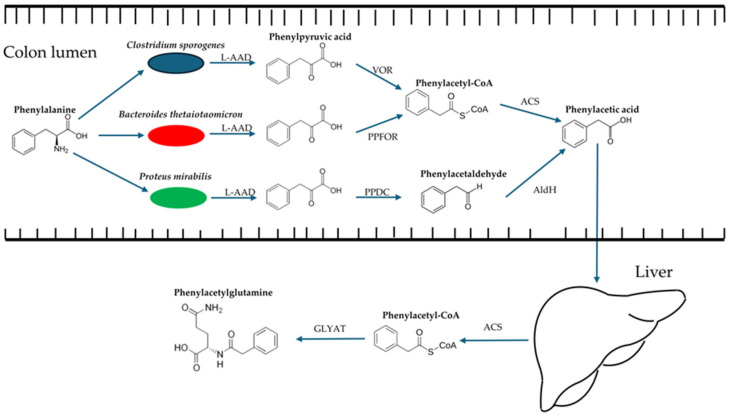
Overview of known PAG biosynthetic pathways. L-AAD (L-amino acid deaminase), VOR (α-ketoisovalerate–ferredoxin oxidoreductase), PPFOR (phenylpyruvate–ferredoxin oxidoreductase), PPDC (phenylpyruvate decarboxylase), ACS (acetyl-CoA synthase), AldH (aldehyde dehydrogenase), GLYAT (glutamine phenylacetyltransferase).

Since then, more studies have further elucidated the mechanisms by which PAG is biosynthesized. Dietary phenylalanine is first converted to phenylpyruvic acid by an L-amino acid deaminase (L-AAD) [208]. Then, the microbial enzyme phenylpyruvate–ferredoxin oxidoreductase (PPFOR) converts it to phenylacetyl-CoA, which is then converted into phenylacetic acid by acetyl-CoA synthetase. A second alternative is where the microbial enzyme phenylpyruvate decarboxylase (PPDC) converts phenylpyruvic acid to phenylacetaldehyde, which is then further converted to phenylacetic acid by aldehyde dehydrogenase. Phenylacetic acid is then absorbed into the portal vein and transported to the liver, where it is conjugated to glutamine or glycine to form phenylacetylglutamine in humans and phenylacetylglycine in rodents, respectively [209]. Furthermore, the abundance of both PPFOR and PPDC in the host gut microbiota correlates with CVD risks. The bacterial species *Bacteroides thetaiotaomicron* is a major contributor to PPFOR, and the species *Proteus mirabilis* is a major contributor to PPDC [210]. Relatedly, it has been observed that the abundance of *Parabacteroides* and *Bacteroides* genera in the gut microbiota correlates inversely with circulating PAG levels, while the abundance of the genus *Escherichia* correlates positively [211]. In addition, further studies support the original *porA* findings with the observation that the abundance of *porA* in the intestinal microbiota is greater in patients with atrial fibrillation compared to healthy controls [212]. Thus, it is likely that numerous microbial pathways exist to produce PAG, many of which have yet to be characterized. The current known PAG biosynthesis pathways are summarized in Figure 3.

In addition to the studies that have already been described, more recent results have associated blood PAG concentrations with incidence of, or negative outcome in, numerous CVDs, including coronary artery disease [213,214], stroke [215,216], type II diabetes [159], type II diabetes with nephropathy [217,218], acute myocardial infarction [159], heart failure [211,219,220,221], atherosclerosis [210], atrial fibrillation [212], in-stent stenosis and restenosis [222,223], and all adverse cardiovascular events [224]. Mechanistically, studies in cell lines have demonstrated the negative impact that PAG has on cellular physiology. In HL-1 mouse cardiac muscle cells, it has been found that treatment with PAG induces apoptosis and increases the amount of ROS [214]. Other results in murine cardiomyocytes indicate that treatment with PAG significantly reduces the pro-contractile effect of epinephrine. In addition, it has been found that treating rat cardio myoblast cells with PAG increases the expression of the *NPPB* gene, which is involved in the production of NT-proBNP. This is known to precede clinically overt changes in left ventricle function and serve as a strong clinical prognostic indicator of mortality. These results have been further confirmed in mice given injections of PAG, which causes a significant elevation in atrial *NPPB* expression [221]. Finally, in a clinical cohort of patients with elevated circulating PAG levels, PAG concentrations have been correlated with the rate of erythrocyte sedimentation and the triglyceride to HDL ratio [223]. Thus, circulating PAG concentrations may be associated with levels of inflammation and dyslipidemia, as well as reduced cardiac muscle contractility and increased cardiac cell stress.

### 3.3. Secondary Bile Acids

The liver biosynthesizes primary bile acids (BAs) in the hepatocytes from cholesterol, generating TCA (taurocholic acid), TCDCA (taurochenodeoxycholic acid), both conjugated to taurine, as well as GCA (glycocholic acid) and GCDCA (glycochenodeoxycholic acid), both conjugated to L-glycine, and finally their non-conjugated versions CA (cholic acid) and CDCA (chenodeoxycholic acid). These are stored in the gallbladder and secreted to the duodenum via the biliary ducts. Once in the intestinal lumen, some of these primary bile acids are deconjugated and further dehydroxylated due to the action of gut microbiota enzymes from the genera *Clostridium*, *Lactobacillus*, *Bifidobacterium*, *Bacteroides,* or *Enterococcus*. This gives rise to secondary BAs, such as DCA (deoxycholic acid), LCA (lithocholic acid), and UDCA (ursodeoxycholic acid), as well as to the primary non-conjugated BAs, CA, and CDCA. About 95% of the BAs that reach the ileum are reabsorbed and recycled in the liver [225]. The hydrophobic BAs, such as LCA, DCA, and CDCA, are cytotoxic for hepatocytes in animal models. The hydrophilic BAs, such as UDCA and the less common TUDCA (tauroursodeoxycholic acid), are however protective [225].

In the intestinal lumen, BAs function as detergents for proper digestion of dietary lipids but also carry out other physiological functions. These include their binding to diverse receptors, such as the farnesoid X receptor (FXR) in the liver and intestine, and the TGR5 (Takeda G protein-coupled BA) receptor in the liver, brain, kidneys, enteric neurons, and skeletal muscle. Binding to the farnesoid X receptor represses genes involved in hepatic BA biosynthesis and inflammation. TGR5 receptor binding enhances energy use in brown adipose tissue and the production of glucagon-like peptide 1 for energy balance and reduces inflammatory effects via NF-κB repression [225,226,227].

Cardiomyocytes, smooth muscle, and endothelial cells also express these same BA receptors. For these tissues, hydrophobic BAs are also toxic, and hydrophilic BAs are also cardioprotective. For example, taurocholic acid (TCA, hydrophobic) causes cardiomyocytes to beat without synchrony (a pathology requiring pacemakers), due to impairment in calcium ion homeostasis. Ursodeoxycholic acid (UDCA, hydrophilic) prevents arrhythmias by stabilizing potassium ion conductance. High BA blood levels, as seen in patients suffering from cholestasis (biliary duct obstruction and impairment of BA secretion to the duodenum), represent a severe risk factor for CVD [228]. Also, the activation of the FXR receptor by some BAs induces the expression of superoxide dismutase, glutathione-S-transferase, and catalase, which reduce oxidative damage in cardiomyocytes [229]. Hydrophobic BAs, such as TCA, cause a prolongation in the QT interval, apoptosis of cardiomyocytes, and ventricular hypertrophy [228].

In this way, diverse BAs have been negatively (CA, TDCA, GCDCA, HDCA, UDCA, LCA) or positively (GCA, DCA, CDCA, GUDCA) associated with CVD parameters, based on metabolomic studies. Of interest is the fact that high GCA serum levels have also been found in patients with biliary disease and liver disease, and high serum DCA (a secondary BA) levels with kidney disease [230]. DCA intravenous administration in animal models for CVD induces hypertension and tachycardia in a dose-dependent manner, whereas exercise reduces its serum levels, both in mice and humans. DCA has some carcinogenic activity, and it has been associated with some cancer types in patients with surgical removal of the gallbladder, which causes DCA levels to increase. These patients also experience a higher incidence of CVD [231]. Patients with liver cirrhosis show high serum BA levels and increased cardiac dysfunction, probably due to reduced fatty acid oxidation (for energy purposes) in cardiomyocytes, mitochondrial damage, and inflammation activation. In vitro, rat cardiac mitochondria suffer altered respiration when exposed to hydrophobic BAs such as LCA, DCA, and CDCA [229].

## 4. Other Metabolites Produced in the Intestinal Environment by Gut Microbiota

Up until this point, we have focused on the small number of molecules that are produced by the gut microbiota and have been directly implicated in CVD prevention or promotion, but these are not the only molecules produced by the gut microbiota. The human gut microbiota has a tremendous metabolic capacity and can produce a huge variety of molecules. Many of them are well known for their impacts on human physiology and can potentially affect the cardiovascular system, either directly or indirectly. Furthermore, it is important to consider these molecules in the context of the gut microbiome itself. Daisley et al. nicely explain the concept of the pantryome, where each species in the gut microbiome is most efficient by exporting its metabolic byproducts and utilizing the metabolic byproducts of others to reduce redundant metabolic processes in the community [94]. From this perspective, it is important to consider that the biosynthesis of a compound by one species can have a direct effect on the abundance and metabolic activity of other species. Thus, metabolites that may not directly affect the human cardiovascular system can influence the activity of other species and induce downstream effects.

In a healthy physiological state, the microbiome is stable, and microorganisms coexist in a cooperative manner, in which they are able to combat stressful situations and avoid significant changes in composition and activity. On the contrary, stressful situations and deregulated responses by the microbiota can trigger the appearance of dysbiosis, which can lead to different diseases, especially of the intestine, such as ulcerative colitis and Chron´s disease [232]. This state indicates a serious metabolic imbalance due to the regulation to which the microbiota is subjected to byproducts generated by its own microbial constituents. These products are, in addition, used as substrates by many other microorganisms. Thus, an imbalance is not only determined by the effect of one particular compound but rather by how that compound can modify the growth or behavior of some bacteria to the detriment of others, to induce a cascading effect on the entire gut microbiota.

It is important to note that the different metabolites produced by some members of the gut microbiota are used as substrates for other neighboring bacteria, so that in a state of health, the production of compounds is perfectly regulated and balanced. An excess of some of these metabolites can become toxic for the host, but high concentrations are prevented as they are metabolized by other microorganisms. Variations in these conditions are harmful and lead to dysbiosis since greater production of some metabolites favors the growth of certain microorganisms to the detriment of others, which can lead to an imbalance in the delicate environment in which they are present. The complexity of these habitats due to inter- and intraspecific relationships, as well as between different domains, reveals that a change in production can trigger a domino effect on the rest of the microorganisms in the matrix, possibly generating systemic diseases. By their nature, gut lumen low-molecular-weight compounds are more easily absorbed since they diffuse into the microbial matrix, so a change in their production easily alters microbiota composition. Ahead there is a brief review of the main gut bacterial metabolites and their influence at a systemic level, considering specifically those molecules with molecular masses lower than 1000 Da [233]. In Table 1, the main gut microbiota metabolites are summarized. It should be noted that many molecules produced by the gut microbiome are likely undetectable or even unknown [234], making their characterization an interesting frontier.

### 4.1. Gases

It is estimated that around 0.2–1.5 L of gases are produced in the human colon every day, with 99% of this volume being H_2_, CH_4_, and CO_2_. Most of these gases pass through the bloodstream and are removed by breathing or by flatulence.

#### 4.1.1. Hydrogen (H_2_)

H_2_ is the most common gas in the intestinal tract, produced by anaerobic fermentation of non-digestible substrates in the colon [101] or through the oxidation of reduced ferredoxin and pyridine nucleotides by hydrogenases [267]. Its amount is highly dependent on the subsequent use of this compound by other microbes, considering that these would have an evolutionary advantage in this ecosystem [235]. Its production is commonly related to *Bacteroidota* phylum and *Ruminococcus* and *Roseburia* genera [101]. Methanogens and sulfate-reducing bacteria use this molecule as a substrate and can compete with each other, which can influence CVD due to its role in maintaining the composition of the gut microbiota [235]. Thus, dysbiosis can trigger an excess concentration of H_2_ varying fermentation processes in the gut that indirectly affect cardiovascular health [101].

#### 4.1.2. Methane (CH_4_)

CH_4_ has no described physiological role and 80% of it is excreted in flatulence and 20% in breath. Its production is mostly related to CO_2_ reduction [268]. Methanogens can be classified into hydrogenotrops, acetotrops, or methylotrops depending on the CH_4_ production pathway used [269]. Gut methane production is associated with certain microbial taxa, including *Bacteroidota* and *Clostridium*, and especially those members of the *Archaea* domain [269,270]. *Methanobrevibacter smithii* is the most significant species in terms of methane production, with around 10^3^–10^9^ CFU/g of this microorganism in the colon [235]. After its microbial production, methane crosses the circulatory system, which is associated with low heart rate and heart failure due to its effect on the parasympathetic nervous system [271,272]. Not all its effects are negative though, with beneficial effects also being described due to its anti-inflammatory and antioxidant properties [273]. Thus, we see the direct impact that gut microbiota dysbiosis can have on cardiovascular health through the dysregulation of methane accumulation.

#### 4.1.3. Carbon Dioxide (CO_2_)

CO_2_ and H_2_ are mainly produced by *Bacillota* and *Bacteroidota*, which constitute over 90% of the gut population [101]. CO_2_ is produced from fermentation of dietary substrates by either conversion of pyruvate to acetyl-CoA or by cleavage of pyruvate to formate, which is subsequently metabolized into H_2_ and CO_2_ by formate hydrogenlyase [101]. Most of the production is absorbed into the bloodstream or utilized by other microorganisms. Although its direct effect on the CSV has not been described, its excess triggers large states of dysbiosis that can trigger indirect harmful effects [235].

#### 4.1.4. Nitric Oxide (NO)

NO is an important signaling molecule, considered to be one of the three gastrotransmitters, since it contributes to regulating immune response, cardiovascular function, blood flow, and metabolism [236]. This compound is not only produced by bacteria by diverse pathways [237] but also by eukaryotic cells through the oxidation of L-arginine by NO-synthase. Some CVDs, such as atherosclerosis, are related to alterations in NO levels, and it is also a known vasodilator [232,274]. Thus, gut microbiota NO biosynthesis may play a significant role in maintaining cardiovascular health.

#### 4.1.5. Ammonia (NH_3_)

NH_3_ is a degradation product from bacterial metabolism and its accumulation is toxic for intestinal cellular integrity, in addition to favoring the growth of certain bacteria to the detriment of others, causing collateral damage to the CVS [236,268]. *Bacteroidota* and *Propionibacterium* are the most common proteolytic taxa [238].

### 4.2. Neurotransmitters

The microbiota has a crucial role in the nervous system. In recent years, the amount of information on the so-called brain–gut–microbiota axis has increased exponentially, presenting evidence of the close interrelation between those elements. This is mainly due to conserved patterns found in certain neurological diseases such as Alzheimer´s or Parkinson’s, where there is evidence of dysbiosis that favors the growth of certain pathogens and decreases beneficial bacteria [238]. Gut microbes produce several neurotransmitters and neuroactive metabolites with different effects, such as excitatory and inhibitory, with some also being biosynthesized by eukaryotic cells. An imbalance in their production can lead to alterations both in the nervous and cardiovascular systems, which might trigger systemic responses. Additionally, bacteria can release signaling metabolites that regulate neurotransmitter production by enteroendocrine cells [236,243,244]. The great influence that the microbiota can have on the production of these molecules is remarkable, even though the greatest physiological production of these molecules resides in nerve cells.

Dopamine is an excitatory neurotransmitter produced by several species of *Bacillus*, as well as by *Serratia marcescens*, *Staphylococcus aureus*, and *Proteus vulgaris*, among others [239]. It is estimated that 50% of dopamine is biosynthesized in the intestine by enteric neurons, intestinal cells, and bacteria; therefore, the microbiota can play a very important role in its regulation [240].

GABA is an inhibitory neurotransmitter produced by several species of *Bifidobacterium* and *Lactobacillus*, among others. It plays an important role in several neurological disorders such as anxiety or depression but also impacts the cardiovascular system by regulating blood pressure and heart rate [240].

Noradrenaline is produced by *Bacillus subtilis*, *Bacillus mycoides*, *E. coli* K-12, and *P. vulgaris*, among others. It is a quorum-sensing molecule, which means that its concentration can generate changes in the gut microbiota due to signaling variations [239].

Serotonin is produced by *E. coli* K-12, *Lactobacillus plantarum*, *Morganella morganii*, and *Streptococcus thermophilus* [243]. This molecule is crucial for various processes such as vasoconstriction, peristalsis, and respiration [240].

### 4.3. Vitamins

The microbes of our gut microbiota are capable of biosynthesizing various types of vitamins for their metabolism, since they are necessary as micronutrients used as cofactors of many essential enzymes. Vitamins can elicit or modulate different pathways, contributing to the maintenance of microbial communities [245]. These essential compounds are supplied by diet or by gut bacterial production. The genera *Bacteroides*, *Bifidobacterium*, and *Enterococcus* are known for producing vitamins, and it is estimated that half of a human’s daily vitamin K is provided by gut bacterial biosynthesis (especially by *Ruminococcaceae*, *Bacteroidota*, *Bifidobacterium*, and *Lactobacillales*) [245,246]. Vitamin K acts as a growth co-factor and has roles in bone and cardiovascular health [245]. Thus, its production levels can have significant effects on the host’s physiology. For example, deficiencies of vitamin B or K can trigger CVD [238]. In addition, deficiency in B group vitamins, such as B1 (thiamin), B2 (riboflavin), B3 (niacin), B5 (pantothenate), B6 (pyridoxine), B7 (biotin), B9 (folate), and B12 (cobalamin), are closely related not only to CVDs but also to other illnesses such as osteoporosis and neuropathy [247]. The *Bacteroidota*, *Fusobacteria,* and *Pseudomonadota* taxa are the main producers of these vitamins with a necessary role in the maintenance of the host’s intestinal homeostasis. It is even estimated that the gut microbiota may provide as much as 30% to 80% of the daily recommended intake for pyridoxine, folate, and cobalamin [94,275].

Vitamin D_3_ deficiency is also associated with hypertension and an increase in vascular tone, which are major risk factors for the development of negative cardiovascular events. It has been described that the gut microbiota regulates the host metabolism of Vitamin D through the production of fibroblast growth factor (FDF). Vitamin D is acquired through diet or produced by the skin after sun exposure [245,248]. Genera, such as *Ruminiclostridium*, *Intestinimonas*, *Pseudoflavonifractor*, *Subdoligranulum*, *Paenibacillus*, and *Marvinbryantia*, which have been associated with hypertension, were shown to be reduced with adequate levels of vitamin D, which suggest its large influence on gut microbiota composition. For this reason, Vitamin D concentration has also been considered a signature indicator of hypertension risk. Vitamin D regulates many physiological processes, determining the expression of several genes involved in response to oxidative stress, apoptosis, and homeostasis as well as cell differentiation, adhesion, and proliferation. Its receptors are located in several tissues, including cardiovascular ones [249].

### 4.4. Amino Acids and Derivatives

The production of amino acids by the gut microbiota plays an important role in the body’s physiology and in maintaining a healthy state in general, while their deficiencies can contribute to the occurrence of severe illnesses and pathologies, such as growth deficiencies [276]. Some types are pointed out: L-isoleucine, L-leucine, and L-valine are the three branched-chain amino acids (BCAAs). Bacteria biosynthesize these amino acids by a conserved pathway that they have in common with eukaryotes, such as plants or fungi, but this pathway is absent in animals, and its production depends on precursor availability [250,251]. They freely circulate into plasma and are incorporated into tissues via specific carriers, serving as building blocks for protein biosynthesis, energy substrates, and signaling molecules [277]. A close relationship between BCAAs and cardiovascular system hazards has been described [278], where high concentrations of BCAAs have been associated with heart failure, stroke, and a higher death rate [279].

#### 4.4.1. Aminovaleric Acid Derivatives

A prominent example of an aminovaleric acid derivative is N,N,N-trimethyl-5-aminovaleric acid (TMAVA or L-carnitine), present in red meat, cod, poultry and cheese. It is a precursor to TMA biosynthesis by the gut microbiota and, thus, to TMAO as well. It is related to heart failure and cardiac hypertrophy due to its implication in the reduction of fatty acid oxidation, which is a hallmark of CVD risk [252]. Similar effects were observed in another derived compound, 5-aminovaleric acid betaine (5-AVAB), present in red meat and milk, also rendering TMA via colon microbiota fermentation [253,254].

#### 4.4.2. Indole Compounds

Indole compounds are generated from non-absorbed L-tryptophan. They are mainly involved in microbial signaling and can be metabolized by gut microbes into tryptamine, skatole, indole, and other indole derivatives such as indole-3-aldehyde, indole-3-acetic acid, indole-3-propionic acid, indole-3-acetaldehyde, indole-3-lactic acid, indole acrylic acid, and indole-3-ethanol [236,255,280,281]. Indole is exclusively produced by bacteria, both Gram-negative and Gram-positive, but in particular by *E. coli*, *P. vulgaris*, *Clostridium* spp., and *Bacteroides* spp. [255].

Indole compounds are partially metabolized in the liver into indoxyl sulfate and other molecules. Some anti-inflammatory, antibiofilm, and bacteriostatic effects have been described in these co-metabolites (resulting from both bacterial and host activity). Indoxyl sulfate is known as a uremic toxin and is considered an indicator of kidney disease. It has been reported to have implications in stimulating oxidative stress and exerting pro-thrombotic and pro-oxidant effects, which trigger cardiac injury, chronic inflammation, coronary artery disease, arrhythmia, and cardiac fibrosis [256]. Therefore, its concentration in blood is determinant to assess the risk of cardiovascular or renal disease. In healthy individuals, it is normally found in low concentrations and bound to proteins, mainly albumin. However, in sick patients, high levels of indoxyl sulfate have been detected [255]. In contrast, indole propionate has been found to be associated with a reduced risk of type II diabetes and left ventricular diastolic dysfunction [12,282]. Other uremic toxins such as indole-3-acetate, TMAO, and the L-phenylalanine derivatives p-cresyl and phenylacetylglutamine are compounds from microbial metabolism of proteins that can trigger CVDs [283,284].

### 4.5. Choline Metabolites

The most relevant choline-derived metabolite is TMA (trimethylamine), or its oxidized form TMAO (trimethylamine oxide), both with important cardiovascular implications that are discussed in Section 3.1. In humans, choline can be oxidized into betaine which can suffer modifications to obtain several derivatives such as dimethylglycine, which is associated with various adverse cardiovascular events, such as acute myocardial infarction and heart failure [257,285]. Other choline-derived metabolites are methylamine and dimethylamine, which are also related to harmful effects on the cardiovascular system [257,258]. In summary, high concentrations of choline metabolites produced by the gut microbiota are a cause of morbidity and mortality, and these data can be used as predictive biomarkers [257].

### 4.6. Fatty Acids and Lipids

The gut microbiota produces many fatty acid compounds. Regarding those with low molecular weight, the most important ones to be biosynthesized by the gut microbiota are bile acids (BAs) and SCFAs, which due to their well-characterized impact on cardiovascular health are discussed further in Section 2.1 and Section 3.3, respectively. Families such as *Sutterellaceae*, *Desulfovibrionaceae*, and *Fusobacteriaceae* increase their relative abundance in high-fat diets while *Christensenellaceae* decreases [286].

#### 4.6.1. Conjugated Fatty Acids

Other compounds that deserve attention are conjugated fatty acids, produced by lactic acid bacteria, from which some isomers (such as conjugated linoleic acid) have been found to have anti-atherosclerotic properties [287]. Branched fatty acids are the predominant fatty acids produced by Gram-positive bacteria and are mainly saturated fatty acids with a methyl branch on the methyl terminal, and they are obtained from the metabolism of branched-chain amino acids. There is a direct relationship between cardioprotective events and branched-chain fatty acids [288], and they have also been described to have anticarcinogenic, anti-inflammatory, insulin sensitivity-reducing, obesity-reducing, and pro-homeostatic properties [287]. These will be extensively discussed in Section 5.3.

#### 4.6.2. Lactate

Another organic acid that is important to mention is lactate. This compound is mostly produced by lactic acid bacteria (LAB) through the glycolysis, pentose phosphate, or bifid shunt pathways, especially of carbohydrates. It acts as an energy source, causes acidification of the environment, and controls the growth of other microorganisms [260,261,262], being involved in multiple physiological processes. Lactate accumulation can lead to neurotoxicity, proliferation of pathogenic bacteria in the gut microbiota, gut disorders, and cardiac arrhythmia [261,262]. In addition, in this situation, the colons’ main colonizing phyla, *Bacteroidota* and anaerobic *Bacillota*, are replaced by *Pseudomonadota*, *Actinomycetota*, and *Lactobacilli* [262,265]. It is important to consider that an excess of lactate supposes not only its accumulation but also the increase in the prevalence of microorganisms that use it, which in turn increases the production of metabolites such as H_2_. This can lead to a rise in concentrations of H_2_S or methane, with the toxicity and systemic implications that this entails having been discussed previously. As a whole, this state of dysbiosis affects cardiovascular health, as previously mentioned.

Heterogeneous groups of microbes can employ lactate as a substrate to generate different products. For example, some *Desulfovibrio* species use it as a co-substrate to produce acetate, while certain *Bacillota* species use it to produce SCFAs. It is even used by some pathogenic genera such as *Campylobacter* or *Salmonella* [262]. This highlights the importance of the regulation of lactate production not only for the microbial ecological balance but also because a change in its composition has the potential to generate a feedback loop by altering the intestinal environment [265].

#### 4.6.3. Lipoteichoic Acid

Lipoteichoic acid is typically produced by members of the *Lactobacillus* genus, especially by *L. plantarum*, which is a well-known probiotic. Lipoteichoic acid is a glycolipid chain that interacts with Toll-like receptor 2 and has been described to have a protective role in immune regulation. These acids modulate the microbial composition as well as various signaling pathways, and their concentration can trigger effects on other systems such as the cardiovascular system [263,264].

#### 4.6.4. Sphingolipids

Sphingolipids can be incorporated by diet or generated endogenously, as well as be produced by members of the *Bacteroidota* phylum using serine palmitoyl-transferase. The presence of sphingolipids influences lipid metabolism, the composition of cell membranes, and intracellular signals. As an example, ceramides promote apoptosis of cardiomyocytes [265,266].

#### 4.6.5. Lipopolysaccharides

These molecules consist of carbohydrate chains bonded to lipid molecules. LPS are considered an innate alarm molecule since they are a structural component of Gram-negative bacteria, so they act as an indicator of pathogen infection. Several portions of LPS are released from bacteria, which provokes different responses due to its immune-stimulant nature, including effects on the cardiovascular system [266]. 

### 4.7. Other Gut Microbiota Metabolites

Zwittermicin (antimicrobial), cereulide (cytotoxic, immunomodulatory), colibactin (cytotoxic), tilivalline (cytotoxic), polyamines (such as spermidine, homospermidine, norspermidine, putrescine, cadaverine, and 1,3-diaminopropane, with multiple roles), or lanthipeptides (ribosomal peptides with important roles in gut homeostasis) [266,267,289,290,291,292,293,294] are just examples of other metabolites that can be produced by the gut microbiota, and many of them are only produced by bacteria. In any case, the variety of compounds produced by the microbiota is enormous and can vary between individuals. Not all metabolites have described effects, much less direct effects on the cardiovascular system, but they can exert systemic effects on other organs, which may end up having indirect impacts on cardiovascular health.

## 5. Dietary Gut Microbiota Modulation for Prevention of Cardiovascular Disease

### 5.1. Probiotics

Probiotics are officially defined as “live microorganisms that, when administered in adequate amounts, confer a health benefit on the host” [295]. Thus, while other sections of this review focus on molecules and their effects on the gut microbiota or host physiology, in this section, we will discuss the use of live microorganisms that are capable of stable engraftment in the gut microbiota and contribute positively to the prevention of CVDs. While, officially, a probiotic must be a pure and well-characterized strain of microorganisms whose health benefits have been demonstrated in clinical studies, the consumption of fermented foods also functions as a less controlled source of potential probiotics for consumers. Both specific strains and fermented foods will be discussed in this section.

The use of probiotics to treat gastrointestinal diseases such as irritable bowel disease, Crohn’s disease, and ulcerative colitis, as well as digestive disorders and even CVDs has been extensively studied. Based on this body of research, recent meta-analyses have become possible. These meta-analyses include a wide range of probiotic microorganisms, with some studies also including foods fermented with defined cultures or even symbiotics containing probiotic cultures along with prebiotic fibers. The results of these studies have been hugely positive, with probiotic treatment being shown to reduce blood pressure, blood cholesterol, blood glucose, body mass index, LDL, insulin resistance, and triglycerides and increase HDL [296,297,298,299,300,301,302]. Furthermore, an analysis of the consumption of fermented dairy products found that consumption reduced the risk of stroke, ischemic heart disease, and cardiovascular death. They also found that probiotic supplements were more effective in preventing CVD when consumed in a dairy matrix rather than in pill format [303]. In contrast to these results, a different analysis found that both probiotic supplements and fermented foods were effective in reducing markers of metabolic syndrome, but only the use of probiotic supplements induced significant effects. The effects of fermented food consumption were not significant [304]. Finally, *Lactiplantibacillus plantarum* is one of the most studied traditional probiotics, but one meta-analysis specifically focused on investigating its effects in CVD prevention found much less favorable results than the other broader analyses. While *L. plantarum* consumption did significantly reduce blood pressure, the effect was not very strong, and there was tremendous heterogeneity among the results [305]. This indicates that the use of mixtures containing multiple probiotic strains is likely to be more effective than the use of individual strains [299].

#### 5.1.1. Dairy Fermenting Probiotics

The most heavily studied and consumed probiotics available today are the traditional dairy fermenting species. Furthermore, in many countries, these are the only bacterial species that can legally be used as probiotics. These generally make up the genera *Lactobacillus*, *Bifidobacterium*, and the species *Streptococcus thermophilus*.

##### *Lactobacillus* 

Studies have shown that *Lacticaseibacillus rhamnosus* consumption decreases inflammation [306] and reduces symptoms of obesity [307]. In addition, extracts of soy milk fermented with *L. rhamnosus* AC1 were proven effective in reducing blood pressure in a hypertensive rat model. This was associated with an increase in blood nitric oxide concentrations, a decrease in blood angiotensin II concentrations, and a potent antioxidant activity as measured in vitro [308]. The fact that this effect was seen without consumption of the live probiotic indicates that the effect is likely mediated by its metabolites. Also, *L. rhamnosus* NCIMB 8010 significantly reduced lipid uptake and prevented the development of insulin resistance in HepG2 hepatic cells [309].

Similarly, *Lactiplantibacillus plantarum* has been shown to reduce markers of inflammation, increase brachial flow-mediated dilation, improve hepatic tissue health, increase fecal lipid concentrations, and increase the abundance of lactic acid-producing bacteria in the gut microbiota [306,310,311]. *L. plantarum* N-1 has been demonstrated as an effective probiotic treatment for CVDs due to its ability to adhere to Caco-2 colon cells, reduce blood cholesterol in hypercholesterolemic rats, and increase fecal butyrate and valerate concentrations [312]. Meanwhile, *L. plantarum* ATCC 14917 has been demonstrated to reduce atherosclerotic lesions as well as markers of oxidative stress and inflammation in aortic tissue [313]. In addition, in hypertensive rats, treatment with *L. plantarum* strains SR37-3 and SR61-2 significantly reduced blood pressure, and this may have been related to the altered expression of genes from the circadian rhythm pathway in vascular tissue [314].

*Lacticaseibacillus paracasei* supplementation has been observed to reduce fasting blood glucose, cholesterol, and triglycerides, while increasing fecal cholesterol, triglycerides, acetate, and propionate [315,316]. The fact that blood cholesterol and triglycerides have been found to decrease while fecal concentrations increase is best explained by the finding that *L. paracasei* strain 201 has been demonstrated to absorb cholesterol when cultured in cholesterol-containing media [316]. Another mechanism by which *L. paracasei* may improve host metabolic health is by altering starch metabolism. This has been demonstrated in healthy adults who consumed bread along with cheese supplemented with *L. casei* 01. They experienced smaller increases in blood sugar levels than those who ate bread alone. Furthermore, in vitro, it has been observed that *L. casei* 01 is effective in inhibiting α-amylase and α-glucosidase activity [317], thus potentially reducing the breakdown of complex starches from the bread into absorbable sugars.

In obese rodent models, *Lactiplantibacillus pentosus* GSSK2 has been found to improve liver health, increase fecal lipid concentrations, and increase the abundance of lactic acid-producing bacteria in the gut microbiota [311]. Meanwhile, *Limosilactobacillus fermentum* PUM has been demonstrated to reduce blood glucose and visceral adipose tissue, while also increasing fecal lipids [311]. Similarly, *Lactobacillus johnsonii* 3121 supplementation in obese mice has proven effective in improving serum lipid profiles and altering the expression of genes related to adipogenesis and lipogenesis in epididymal white adipose tissue [307]. While these probiotics have functioned by decreasing intestinal lipid uptake, as demonstrated by increased fecal lipids, *Limosilactobacillus reuteri* CCFM8631 has been proven to function by reducing blood TMAO concentrations in atherosclerotic mice, while also increasing the concentrations of fecal SCFAs [318]. In an entirely separate mechanism, the cell-free extract of *Lactobacillus acidophilus* NX2-6 has been seen to protect HepG2 cells from mitochondrial, oxidative, and endoplasmic reticulum stress when exposed to oleic acid [319]. This once again indicates the protective effect of metabolites produced by this strain.

##### *Bifidobacterium* 

*Bifidobacterium longum* BB536 supplementation in hypercholesterolemic adults has been demonstrated to modify blood lipid biomarkers associated with reduced visceral adipose tissue and elevated HDL [320]. Furthermore, *B. longum* has been shown to reduce the conversion of choline to TMA and, thus, to reduce the blood concentration of TMAO, by reducing the abundance of genera that are known to contain the *cut* gene cluster that converts choline to TMA [321]. A similar effect has been observed with *Bifidobacterium breve* [321]. Furthermore, *B. breve* CECT7263 has been shown to reduce blood pressure in a similar way to treatment with butyrate and acetate, indicating that their increased biosynthesis is a likely mechanism of action.

*Bifidobacterium animalis* has also demonstrated very promising properties. Consumption of yogurt supplemented with *B. animalis* dn-173010 has been shown to reduce fasting blood glucose and increase blood HDL concentrations [322]. Similarly, *B. animalis* 01 and *B. animalis* subsp. *lactis* GCL2505 have both been demonstrated to be effective in preventing diabetes and liver damage [323], as well as dietary-induced obesity. Crucially, in mice with GPR43 knocked out, which is a known SCFA receptor, treatment with this species had no effect on obesity [324], indicating that SCFA production is a likely mechanism by which these positive effects are induced. Similarly, *Bifidobacterium pseudolongum* supplementation has been demonstrated effective in reducing hepatic fibrosis, while the effects of the probiotic treatment were almost identical to the effects of direct butyrate supplementation [325].

#### 5.1.2. Other Potential Probiotics

Other bacteria that have been studied as potential probiotics include the genera *Bacillus*, *Enterococcus*, and *Pediococcus*. From the *Bacillus* genus, *Bacillus amyloliquefaciens* has been demonstrated to increase glucose tolerance in mice with a microbiota-independent effect, potentially by inducing an increase in hepatic and intestinal glucose uptake as observed in cell lines. Furthermore, simple dietary ingestion of the strain’s exopolysaccharides was sufficient to achieve this result [326]. In addition, in mice with high-fat dietary-induced obesity, treatment with *Bacillus licheniformis* Zhengchangsheng significantly reduced body weight, body fat percentage, blood glucose, and cholesterol markers, as well as hepatic inflammation and hepatic expression of lipogenesis-related genes [327].

From the *Enterococcus* genus, *Enterococcus faecium* 132 has been demonstrated to take up cholesterol when cultured in cholesterol-containing media and effectively reduce blood and hepatic cholesterol concentrations while increasing fecal cholesterol in a hypercholesterolemic rat model [326]. Meanwhile, *Enterococcus faecalis* AG5 effectively reduced body weight, blood cholesterol, triglycerides, insulin, and leptin, as well as adipocyte hypertrophy and fatty acid accumulation in obese mice [328].

From the *Pediococcus* genus, which is heavily present in fermented foods, *Pediococcus acidilactici* NCIMB 8018 was demonstrated to reduce lipid uptake and prevent the development of insulin resistance in HepG2 cells [329], while *Pediococcus pentosaceus* KID7 effectively reduced body weight, white adipose tissue, and blood and hepatic cholesterol in an obese mouse model [307].

A special mention must be given to *Akkermansia muciniphila* since it has gained tremendous attention in recent years due to its association with numerous health benefits [329]. In a clinical cohort of adults with dyslipidemia, probiotic treatment with *A. muciniphila* significantly altered the concentrations of blood metabolite biomarkers [330]. In a separate study in rats with atrial fibrillation, a reduction in the intestinal abundance of *A. muciniphila* correlated with increased TMA and TMAO as well as increased symptoms of cardiac fibrillation. In continuation, probiotic treatment with *A. muciniphila* reduced TMA, TMAO, and cardiac fibrillation symptoms [331]. Of further interest is the finding that cold exposure decreases the abundance of *A. muciniphila*, meaning that supplementation with this probiotic species may be of interest to people in colder climates or who regularly experience colder conditions.

#### 5.1.3. Genetically Modified Probiotics

While genetically modified strains are likely to have difficulty achieving approval for human consumption due to bans on genetically modified organisms in some countries, they offer the opportunity to augment the already beneficial effects of individual probiotic strains. One study compared the effects of wild-type *L. reuteri* and a strain that was engineered to secrete IL-22 in a diet-induced obese mouse model. In agreement with the previously described results, they found that treatment with both strains yielded reduced body weight and reduced fat pad area. Of great excitement is the further finding that the modified strain also reduced liver weight and triglyceride concentrations, while the wild-type strain had no effect on these [332]. Further research is ongoing to engineer probiotics that can sense the concentration of SCFAs in the gut lumen and respond in desirable manners, which could be beneficial for a wide range of diseases, including cardiovascular ones [333].

#### 5.1.4. Probiotic Mixtures

Due to all the positive results described, as well as the previously described observation that probiotic mixtures containing multiple strains tend to be more effective than treatments with individual strains, numerous recent studies have investigated the use of probiotic mixtures containing a wide variety of strains to treat CVDs. Two studies using commercially available mixtures found that their treatments effectively reduced body weight, fat mass, and blood cholesterol and glucose markers in obese murine models. One of the studies also found that treatment reduced hepatic steatosis and improved vasoconstriction while increasing nitric oxide release from mesenteric segments [334]. The other study found that treatment also reduced adipose tissue inflammation and natural killer T-cell depletion. Furthermore, CD1d-knockout mice that lack adipose natural killer T cells experienced more severe obesity and reduced benefits from probiotic treatment [335]. This brings into perspective the involvement of the immune system in systemic obesity as well as the immune-modulating effects of the gut microbiome. Further research into how probiotics can impact this axis is greatly warranted. Finally, in a third study, the authors designed their own probiotic mixture by selecting strains that are particularly exceptional at glucose metabolism in vitro. Mice treated with this mixture experienced significantly reduced blood glucose concentrations after undergoing a glucose tolerance test. Furthermore, diabetic mice treated with this mixture experienced reduced fecal and blood glucose levels [336]. Thus, treatment with sugar-metabolizing bacteria may be effective in reducing host glucose uptake.

#### 5.1.5. Fermented Foods

While a great deal of research has gone into studying probiotics as well-defined strains, traditionally, humans would have been exposed to potential probiotic microorganisms via fermented foods. Even today, these foods are a significant part of many human diets and as such, their impact on the gut microbiota and cardiovascular health should be considered. In a model of hypertensive rats, dietary kefir supplementation significantly reduced mean arterial pressure, intestinal permeability, and inflammation in the hypothalamic paraventricular nucleus [337]. In a separate clinical study of patients with metabolic syndrome, kefir consumption significantly decreased blood pressure, fasting blood glucose, blood cholesterol, and Framingham scores [337]. In contrast, a study of prediabetic adults found that natural yogurt consumption did not affect fasting blood glucose levels but did reduce circulating glycosylated hemoglobin levels [338]. Furthermore, a study in adults with metabolic syndrome found that kefir consumption did reduce blood pressure and LDL concentrations, but the same effect was also found with the consumption of non-fermented milk [339]. Thus, the results seem promising, but more research is needed to confirm these findings. Furthermore, studies would benefit from a deeper characterization of the microorganisms present in these naturally fermented foods. In addition, as it has been commented previously in this section, the metabolites produced from microbial fermentation can induce beneficial effects. Thus, a deep chemical characterization of these fermented foods should be conducted to elucidate the bioactive compounds that are found in them.

#### 5.1.6. Remaining Questions

While the majority of results indicate the promise of probiotic treatment to promote cardiovascular health, some studies have concluded conflicting results, and some questions remain. Two clinical studies using probiotic mixtures found no evidence of efficacy. In one study, patients with nonalcoholic steatohepatitis experienced no change in blood lipid or glucose parameters or body mass index [340]. In the second, obese diabetic patients experienced no change in biomarkers of blood glucose metabolism, but they did experience reduced hip circumference and reduced intestinal permeability as measured by reduced blood zonulin concentrations [341]. In addition, a study of prediabetic patients treated with *L. rhamnosus* HN001 along with intermittent fasting found that probiotic treatment did not induce any further improvements in cardiovascular health beyond those induced by intermittent fasting alone [342]. This brings into question whether the positive effects of probiotic treatment will continue to be relevant when modifications in diet and physical activity are implemented first. Further confirming this is the observation that hypercholesterolemic patients treated with dietary modifications and fecal microbiota transplants from a lean donor experienced no improvement in blood lipid profiles beyond those induced by the dietary modification alone [343]. Within the broad umbrella of diseases that encompass CVD, some may be more difficult to treat with lifestyle changes and thus could benefit more from probiotic treatment.

In addition, a separate study treating hypercholesterolemic adults with a probiotic mixture and berberine, a plant metabolite, found that probiotic treatment alone had no effect on blood lipid profiles while co-treatment with berberine did. This was further explained by the observations that *Bifidobacterium breve*, when cultured in lipid-containing media in vitro, exhibited increased lipid uptake in the presence of berberine [344]. Thus, the exact activity of probiotic strains in the digestive tract needs to be further elucidated so that this activity can be modulated to achieve maximum results.

### 5.2. Prebiotics

A prebiotic is defined by the International Scientific Association for Probiotics and Prebiotics (ISAPP) as “a substrate that is selectively utilized by host microorganisms, conferring a health benefit”. These substances typically have a plant origin and are characterized by their resistance to degradation by human digestive enzymes. As a result, they remain stable until they reach the colon, where they are utilized by a limited number of intestinal bacteria, promoting their growth and producing bioactive compounds such as SCFAs through fermentation by the gut microbiota [20,345]. The primary prebiotics are predominantly composed of oligosaccharide chains with varying lengths and degrees of branching. Key examples include inulin, fructo-oligosaccharides (FOS), galacto-oligosaccharides (GOS), lactulose, and human milk oligosaccharides (HMO) [346].

Numerous studies suggest that the use of prebiotics in the diet may serve as a protective mechanism against various diseases that can lead to CVD. This is primarily due to the selective growth of beneficial gut microbiota and the production of anti-inflammatory and bioactive molecules such as SCFAs (see Section 2). In this context, it has been postulated that fiber-rich diets may have beneficial effects due to the increased concentration of SCFAs, which can lead to a reduction in blood pressure, limited cardiac hypertrophy, and lower ventricular dilatation. Additionally, these prebiotic-rich diets are capable of reconstituting the altered microbiota observed in conditions such as heart failure and improving the integrity of the intestinal barrier [347,348]. Similarly, a meta-analysis demonstrates that prebiotics, particularly inulin-type fructans, may confer protection against CVD risk by reducing plasma levels of LDL-C, triacylglycerides, and body weight. This protective effect is particularly evident in populations with a high risk of obesity, provided that prebiotics are consumed consistently for at least 6 weeks [349].

One of the primary SCFAs implicated in CVD is acetate. Dietary intake of inulin markedly elevates the levels of this SCFA, thereby activating its receptors in the liver, particularly free fatty acid receptor 2 (FFAR2). This activation modulates the production of critical molecules such as glucagon-like peptide-1 (GLP-1) and peptide YY (PYY). Dysregulation of GLP-1 and PYY is associated with an increased risk of hypertension, atherosclerosis, cardiac arrhythmias, and heart failure [28,76].

These findings are corroborated by various clinical trials. For instance, one study in elderly individuals demonstrated that supplementation with a symbiotic mixture containing *Lactiplantibacillus plantarum* PBS067, *Lactobacillus acidophilus* PBS066, and *Limosilactobacillus reuteri* PBS072, plus prebiotic fibers (inulin and FOS), effectively reduced visceral adipose tissue, blood LDL, triglycerides, fasting insulin and glucose, and mean arterial pressure due to the reduction in serum levels of high-sensitivity C-reactive protein (hsCRP) and TNF-α [350].

Interestingly, hypercholesterolemic adults who were prescribed dietary fiber supplementation (14.6 g of dietary fiber/day for 2 months) experienced reduced blood LDL levels, and those participants who did not respond to the fiber treatment had significantly less abundance of SCFA-producing genera in their fecal microbiotas. Furthermore, fecal propionate levels post-treatment correlated inversely with blood LDL levels [56]. In this sense, a study in obese adults found that treatment with a symbiotic blend containing *L. casei*, *L. rhamnosus*, *L. acidophilus*, *L. bulgaricus*, *B. longum*, *B. breve*, and *S. thermophilus* with FOS as a prebiotic fiber was very effective in reducing fasting blood glucose levels [351].

Similar results in CVD were observed in a study involving women with type II diabetes, who were supplemented with 10 g/day of high-molecular-weight inulin for 8 weeks. This study demonstrated a reduction in inflammatory markers and metabolic endotoxemia through multiple pathways: a decrease in body weight, reduced levels of lipopolysaccharide due to enhanced intestinal integrity, and lowered oxidative stress levels [352,353].

Promising results were also observed in a study involving hemodialysis patients, for whom CVD is the leading cause of death. The administration of a symbiotic formulation containing 5 g of FOS, GOS, and inulin resulted in a significant reduction in the expression of soluble intercellular adhesion molecule-1 (sICAM-1), an endothelial cell adhesion marker. This reduction was even more pronounced in the patient groups receiving the symbiotic compared to the group receiving only the probiotic strains included in the symbiotic. Therefore, the addition of prebiotic fibers appears to exert a protective effect in these patients. This effect may be attributed to a decrease in systemic inflammation, as pro-inflammatory cytokines are mediators of the activation and expression of molecules such as ICAM-1 and VCAM-1. In patients supplemented with the symbiotic, these pro-inflammatory cytokines were found at reduced levels [354].

Based on data supported by meta-analyses and cohort studies, high dietary fiber intake is estimated to be associated with a 26% reduction in CVD-related mortality. Furthermore, additional studies indicate that an increase of 7 g/day in fiber intake is correlated with a 7% reduction in the risk of hemorrhagic and ischemic stroke [355,356].

The findings on the role of prebiotics in the prevention of CVD are highly promising. However, these data should be interpreted with caution due to several factors that could confound the conclusions, including the variability in study designs and execution among clinical trials, as well as the challenge of accounting for all variables in the complex diets of individual participants. Consequently, more standardized and rigorous studies are needed to validate the positive correlation between prebiotic consumption and protection against CVD.

### 5.3. Lipids

Lipids have essential functions as structural components, signaling molecules, and sources of energy. Specifically, only the group known as functional lipids has been defined with important functions in microbiota modulation, as well as offering multiple benefits against several diseases [357,358]. These compounds have a crucial role in cardiovascular health as they are responsible for preventing the oxidation of proatherogenic molecules and down-regulating the expression of inflammatory proteins. Lipids can be classified into several groups depending on their properties. Although high concentrations of these molecules cause serious problems at the cardiovascular level, all of them present beneficial effects for the cardiovascular system at optimal concentrations. Furthermore, the synergistic effects of several functional lipids have been described as being positive for the prevention of CVDs [359].

CVDs are mostly prompted by risk factors that can be avoided. Hyperlipidemia, high blood pressure, lipid disorders, obesity, diabetes, and sedentarism are some of the factors that principally determine cardiovascular risk, and statins are one of the most frequent drugs used for their treatment. However, many patients develop negative side effects, so other therapies such as the use of nutraceuticals are of great interest. The consumption of monounsaturated and polyunsaturated fats to the detriment of saturated ones has been shown to be an important modulator that reduces the risk of CVD [360,361]. Associations between fat intake and cardiovascular risk have been extensively investigated.

#### 5.3.1. Monounsaturated Fatty Acids (MUFAs)

MUFAs structurally present only one double bond and can be found in seeds, olives, peanuts, and breast milk [352,362]. They are associated with an increase in the *Bacteroidota*/*Bacillota* population ratio, which is normally associated with greater production of SCFAs and all the benefits that this entails, as well as reduced weight gain, hypertension, insulin resistance, and obesity. In addition, MUFA’s apolipoproteins present a high affinity for hepatic receptors and activate several triacylglycerol metabolism pathways [357,362]. These lipids present clear implications for improving lipid profiles and reducing cardiovascular risk factors such as obesity or hypertension [363]. Consumption of omega-9 fatty acids such as oleic acid, found primarily in olive oil and some meats, has been found to induce anti-inflammatory, antioxidant, and cardioprotective effects [364,365]. Omega-7 fatty acids (double bond in the seventh position) found in plant oils do not present as much interest nor have they been as exhaustively studied as other types of monounsaturated fatty acids, but antioxidant and anti-inflammatory effects have been described [366]. The omega-7 palmitoleic acid is a major product of endogenous lipogenesis, especially in the liver. It is present in fish and vegetable oils, participates in the maintenance of homeostasis, and is also related to insulin sensitivity and glucose tolerance [367]. There is an inverse relationship between red blood cell vaccenic acid (an omega-7 fatty acid) and the risk of heart failure [368]. Other types of monounsaturated acids have not been studied as extensively as polyunsaturated ones.

#### 5.3.2. Polyunsaturated Fatty Acids (PUFAs)

PUFAs contain between two and six double bonds [357], and although they are crucial to health maintenance, they cannot be biosynthesized de novo and must be incorporated through diet [360]. They are crucial for the brain and heart’s normal functioning, and different studies have demonstrated that their consumption is key to reducing adverse cardiovascular events [360]. It is important to highlight that although they are compounds that are described as beneficial, it is essential to maintain a proper balance of omega-3/6 fatty acids since an alteration can trigger inflammatory events [360]. They are classified depending on their double-bond position, and their consumption is associated with decreased obesity, weight gain, inflammation, and diabetes, as well as multiple cardiometabolic benefits [357]. 

##### Omega-3 Fatty Acids

Omega-3 fatty acids reduce the incidence of stroke, arrhythmia, atherosclerosis, inflammation, and blood pressure. They are found in nuts, vegetable oils, and eggs. Deficiency in these polyunsaturated fatty acids is closely tied to the development of vision abnormalities [357,360]. Individuals with coronary heart disease benefit from the consumption of omega-3 fatty acids, with a good dose estimated to be around 0.5–1.8 g per day [358]. Alpha-linolenic acid is a precursor of other omega-3 fatty acids, since it can be converted by enzymatic action into eicosapentaenoic acid (EPA) and docosahexaenoic acid (DHA). It can be found in seeds, vegetal oils, and nuts and is mainly acquired by dietary intake [357,369], constituting the principal polyunsaturated acid in the diet. When the concentration of this compound becomes elevated, it can be transformed into other types of polyunsaturated acids or other structural lipids [357]. Although the studies on this compound are not as extensive as for its derivatives, there is evidence that points to protective effects on the cardiovascular system [370]. A clinical study showed that supplementation with EPA, commonly found in seafood [360], dramatically reduces carotid intimal-medial thickness, implying the reduction of atherosclerosis and enhancement of endothelial function [357]. DHA, also present in seafood [360], participates in the reduction of LDL-cholesterol levels and mediates other antioxidant and anti-inflammatory processes [369].

##### Omega-6 Fatty Acids

Omega-6 fatty acids are found in soy, nuts, pork, butter, and corn [360]. They have an important role as structural components, modulate membrane function, and serve as precursors of eicosanoid acids that modulate vascular tone and inflammatory responses [357]. Clinical studies have demonstrated that consumption of omega-6 fatty acids is related to cardiovascular risk reduction and improved long-term insulin resistance [357]. Linoleic acid deficiencies are related to fatty liver disease and poor growth [360]. The conjugated version of linoleic acid, present in dairy products, also has other important physiological properties such as being anticarcinogenic, anti-obesogenic, anti-atherosclerotic, and favoring lipolysis and fat burning [362].

On the other hand, arachidonic acid, another polyunsaturated omega-6 fatty acid, is a constituent of the lipid bilayer of cell membranes and is released mainly by phospholipase A2 but also by D and C versions [371]. It can be acquired from meat (chicken, beef, and pork), eggs, and breast milk, as well as being especially present in fish due to its role in growth and reproduction [372], but it also can be obtained by the conversion of linoleic and gamma-linoleic acids. It is estimated that 5–10% of linoleic acid incorporated into membranes is later transformed into arachidonic acid. Its presence in breast milk is because of its important function in growth and nervous system development, which is why it is also added to milk formula [372]. Arachidonic acid is metabolized by different enzymes such as w-hydroxylases and epoxygenases of the cytochrome P_450_ family, the cyclooxygenase (COX) and lipid oxygenase pathways (LOX), and in several tissues such as the kidney, heart, liver, and lungs [372]. Depending on the enzymes and tissues, arachidonic acid can be transformed into several different metabolites, each one exerting different roles in the prevention of CVD by binding to tissue receptors that trigger different signaling pathways. Free forms of this compound are a minority but can be subjected to the effects of reactive oxygen and nitrogen species, generating products such as isoprostanes and nitroeicosatetraenoic acids that generate harmful effects on health, such as platelet aggregation and cardiomyocyte hypertrophy [371]. Besides being a structural component, it has many other functions such as being a regulator of gene expression and modulator of inflammation and ion channel fluxes, and it has an essential involvement in the development of obesity, diabetes, and fatty liver disease [371]. Prostaglandins, thromboxanes, prostacyclins, hydroxyecosatetraenoic acids, leukotrienes, lipoxins, hypoxins, and anandamides are some of the products of arachidonic acid metabolism, each with different properties [371].

#### 5.3.3. Medium Chain Fatty Acids

Caproic acid, caprylic acid, capric acid, and lauric acid are medium-chain fatty acids that naturally occur esterified to a glycerol backbone, giving rise to medium-chain triglycerides. These forms are more easily absorbed than long-chain fatty acids. They are mainly found in coconut oils, palm oil, and dairy products. They are also incorporated in newborn formula since their properties contribute to a reduction in fat deposition and, consequently, a reduction in steatogenic illnesses [357,373]. They serve as an immediate energy source and they control obesity by inducing an increase in fat oxidation and energy consumption. Furthermore, they have been demonstrated to be beneficial for psychological health, social behavior, and stress reduction, as well as exhibiting some antimicrobial activity [373].

#### 5.3.4. Phytosterols

Phytosterols are mainly found in nuts, vegetables, and their oils. Betasitosterol, campesterol, and stigmasterol are the most abundantly consumed. These are cholesterol-like molecules but more hydrophobic, having a higher affinity for fat-digesting micelles than cholesterol, which reduces its absorption in the intestine. Thus, their supplementation decreases the level of cholesterol absorption, with the consequent positive implications that this has on cardiovascular health [357]. In addition, phytosterols have anticarcinogenic and inflammation-modulating properties [374].

#### 5.3.5. Vitamin E

Vitamin E and its derivatives, tocopherols and tocotrienols, are plant-derived molecules consisting of a phytyl chain and a chromanol ring. They are found in nuts and vegetable oils. Vitamin E has been described as an antioxidant due to its capacity to eliminate chain oxidative reactions and lipid radicals [375]. It has also been described as anti-inflammatory and anti-atherothrombotic due to its platelet aggregation-reducing properties. Finally, it is also a modulator of signaling pathways [376].

#### 5.3.6. Diacylglycerols

Diacylglycerols are associated with a reduction in obesity and adverse cardiovascular events since they decrease the levels of C-reactive protein (CRP), tumor necrosis factor-alpha (TNF-α), fat accumulation, and platelet aggregation, which are considered CVD risk factors. Diacylglycerols are found in rice and sunflower seeds, and their supplementation has been shown to induce a decrease in triacylglycerols and cholesterol levels in serum [377]. As a whole, they prevent obesity and fat accumulation with the consequent effects on the cardiovascular system.

#### 5.3.7. Carotenoids

Compounds belonging to the carotenoid group are constituted by 8 units of isoprenoids (C5 building blocks) and consist of more than 700 molecules in nature, classified as xanthophylls and carotenes. These molecules are classified as unsaponifiable lipids within the structural family of terpenes. They are mainly present in fruits and vegetables and exert enormous antioxidant activities. They have been found to prevent the oxidation of plasma proteins, which is associated with a reduction in atherogenic events [377,378]. Lycopene is the most abundant carotenoid in blood and it is considered fundamental to the cardiovascular risk-reducing impact of the Mediterranean diet, reducing strokes and mortality rates [378]. Similarly, astaxanthin, found in seafood and microalgae, has demonstrated protective effects against metabolic syndrome in rodent models [379] as well as improving cardiac function in heart failure patients [380]. In contrast, β-carotene supplementation has been demonstrated to be effective in reducing markers of metabolic syndrome in rodent models [381,382], but meta-analyses of human trials have associated it with an increased risk of CVD [383,384].

### 5.4. Polyphenols

Polyphenols include a large family of nutraceuticals, with major sub-families including flavonoids (such as quercetin from apple), phenolic acids (such as 4-hydroxytyrosol from extra virgin olive oil), lignans (such as secoisolariciresinol diglucoside from flax seeds), and tannins (such as punicalagin from pomegranate). All of them contain hydroxyl moieties bound to aromatic rings in diverse numbers and configurations and are plant secondary metabolites with interesting bioactivities. Most of them reach the colon, where gut microbiota metabolism generates low-molecular-weight derivatives that are absorbed to the portal vein and transferred to the liver, modified (via methylation, sulfation, etc.), and from there, circulated to other tissues [385].

Polyphenols have been shown to induce a reduction in blood pressure, potentially by modulating NO (a known vasodilator) production, which is considered a cardioprotective mechanism [386]. High dietary flavonoid consumption has been associated with a lower risk of CVD and metabolic syndrome parameters (high-serum triglycerides, low-serum HDL cholesterol, hypertension, and high-serum glucose) [386].

Among flavonoids, the flavanols present in *Camellia sinensis* tea, especially (-)-epigallocatechin-3-gallate (in comparison with (-)-epigallocatechin, (-)-epicatechin-3-gallate, and (-)-epicatechin), are particularly promising. They have been demonstrated to have hypotensive, antioxidant, anti-inflammatory, anti-proliferative, anti-thrombotic, and lipid profile-modulating activities. All of these are of interest in CVD prevention. Hypotensive mechanisms include inhibition of the renin–angiotensin II–aldosterone system and the activation of NO biosynthesis. Antioxidant mechanisms include radical scavenging activity, as well as Nrf-2 and superoxide dismutase activation. Anti-inflammatory mechanisms include reduction of C-reactive protein, inhibition of cytokines such as NF-κB and TNF-α, as well as inhibition of neutrophil migration to artery walls. Anti-proliferative mechanisms include inhibition of G1- to S-phase transition in vascular smooth muscle cells and inhibition of matrix metalloproteases. Anti-thrombotic activity includes inhibition of platelet aggregation via inhibition of collagen-mediated phospholipase C-γ2, blockade of protein tyrosine phosphorylation, and the enhancement of calcium ATPase activity. Finally, lipid-modulating activities include the reduction of serum triglycerides, LDL cholesterol, and increased HDL cholesterol [387]. The specific role of flavonoids as inhibitors of the NF-κB pathway is of great interest, as this transcription factor regulates the expression of hundreds of genes involved in inflammation, immunity, cell proliferation, and apoptosis [388]. Quercetin (orally at 10 mg/kg/day) reduces the activation of NF-κB via the toll-like receptor pathway in rats and also the activity of matrix metalloprotease, downregulating the coronary hypertrophic remodeling associated with hypertension [388]. Other flavonoids, such as icariside (a monoglucosylated metabolite of the prenylated plant flavonoid icariin, from *Epimedium grandiflorum*) and morin (a flavonoid from guava fruit, *Psidium guajava*), inhibit the NF-κB pathway via the downregulation of IL-1β serum levels, a pro-inflammatory cytokine [388].

## 6. Concluding Remarks

Over the last two decades, the advent of next-generation sequencing and metagenomics technologies has led to an explosion in research involving the gut microbiome [389,390,391]. While originally these studies were largely based on metagenomic sequencing data, more recent studies (such as those discussed in this review) have combined these metagenomic interrogations with metabolomics, as well as other compound discovery techniques. This has led to a more mechanistic understanding of how the gut microbiome impacts host physiology, including cardiovascular health. While a large number of compounds have been characterized and dietary strategies to modulate the biosynthesis of these compounds have been explored, truly direct and controlled modulation of the gut microbiome is a long way off. Studies are currently underway in highly controlled and defined bacterial communities to understand the impact of specific species on the whole community’s metabolic output [392]. Work such as this would benefit from the development of a stable in vitro synthetic microbiome that accurately models the human gut microbiome. Furthermore, the development of genetic engineering tools that can effectively and specifically modify the genomic landscape of the entire community would give researchers a tremendous level of control over the metabolic outputs of said community [393]. Finally, while the majority of microbiome research has focused on the bacterial fraction of the community, the importance of the other fractions, such as the archaea, virome, and mycome, is only beginning to be understood [394,395]. In addition, as previously stated, many (if not the majority) of the chemical compounds produced by the gut microbiome are entirely unknown. Only continued research into the chemical space of the gut microbiome will bring more of these compounds to light. Thus, the scientific community has identified many bacterial species associated with CVD disease as well as many compounds produced by these species that can directly or indirectly affect CVD. In the coming years, we may learn to engineer the gut microbiome in a more robust and specific manner, characterize the impact of its non-bacterial members, and continue to identify more of the chemical space in which the members of the gut microbiome live.

## Figures and Tables

**Table 1:** Main microbiota metabolites.

Compounds	Mainly Produced by	Activity	References
H_2_	*Bacteroidota* phylum, *Ruminococcus* spp., *Roseburia* spp.	Maintains the community structure of gut microbiota	[101,235]
CH_4_	*Bacteroidota* phylum, *Archaea* domain *(Methanobrevibacter smithii)*, *Rhodopseudomonas palustris*, *Clostridium* spp.	Anti-inflammatory and antioxidant properties	[101,231,232,233,234]
CO_2_	*Bacillota* phylum, *Bacteroidota* phylum	Acetogens and methanogens reduce CO_2_ to produce acetate and methane, respectively	[101,235]
NO	*Escherichia coli*, *Salmonella typhimurium*, *Bacillus subtilis*	Regulates immune response, cardiovascular function, blood flow, and metabolism	[236,237]
NH_3_	*Bacteroides* spp., *Propionibacterium* spp.	Degradation product, precursor molecule	[101,236,238]
Dopamine	*Bacillus* spp., *Serratia marcescens*, *Staphylococcus aureus*, *Proteus vulgaris*	Proper maintenance of immune response and brain functioning	[239,240]
Glutamate	*Lactobacillus* spp., *Bifidobacterium* spp., *Corynebacterium* spp.	Interkingdom signaling and modulation of physiological conditions	[241,242]
Noradrenaline	*Bacillus subtilis*, *Bacillus mycoides*, *P. vulgaris*	Stimulates *quorum sensing*, signaling molecule	[239]
GABA	*Bifidobacterium* spp., *Lactobacillus* spp.	Regulates blood pressure and heart rate	[240,241,243]
Serotonin	*E. coli*, *Lactiplantobacillus plantarum*, *Morganella morganii*, *Streptococcus thermophuilus*	Regulates nervous system, vasoconstriction, peristalsis, and respiration	[240,241,243]
Acetylcholine	*L. plantarum*, *Bacillus* spp.	Regulates intestinal motility and enteric neurotransmission	[244]
Histamine	*Citrobacter freundii*, *Enterobacter* spp., *Lactobacillus* spp., *Lactococcus* spp., *Pediococcus parvulus*	Regulates immune system	[244]
Vitamin B	*Bacteroidota* phylum, *Fusobacteria* phylum, *Pseudomonadota* phylum	Regulates many physiological processes, maintenance of host’s intestinal homeostasis	[245,246,247]
Vitamin K	*Ruminococcaceae* family, *Bacteroides* spp., *Bifidobacterium* spp. *Lactobacillales* order	Regulates many physiological processes, co-factor for growth, role in bone and cardiovascular health	[245,246]
Vitamin D_3_	*Ruminiclostridium* spp., *Intestinimonas* spp., *Pseudoflavonifractor* spp., *Subdoligranulum* spp., *Paenibacillus* spp., *Marvinbryantia* spp.	Regulates many physiological processes, expression of several genes of oxidative stress, apoptosis, homeostasis, cell differentiation, adhesion, and proliferation	[245,246,248,249]
Branched amino acids (BCAAs)	*Lentilactobacillus buchneri*	Building blocks for protein biosynthesis, energy substrates, or signaling molecules	[250,251]
Amino valeric acid derivatives	*Clostridium* sp.	Reduction of fatty acid oxidation, reduction of CVD risk	[252,253,254]
Indole derivatives	*E. coli*, *P. vulgaris*, *Clostridium* spp., *Bacteroides* spp.	Ligands for aryl hydrocarbon receptors, an indicator of kidney disease, stimulate oxidative stress, and exert pro-thrombotic and pro-oxidant effects	[255,256]
Choline derivatives	*E. coli*, *Citrobacter* spp., *Klebsiella pneaumoniae*, *Shigella* spp. *Achromobacter* spp., *Sporosarcina* spp., *Actinomycetota* phylum	Atherosclerosis biomarker	[257,258,259]
Lactate	*Lactobacillus* spp., *Leuconostoc* spp., *Pediococcus* spp., *Lactococcus* spp., *Streptococcus* spp.	Regulates physiological processes	[260,261,262]
Lipoteichoic acid	*Lactiplantibacillus plantarum*	Role in immune regulation, inflammatory processes	[263,264]
Sphingolipids	*Bacteroidota* phylum	Regulates lipid metabolism, composition of cell membranes, and intracellular signals	[265,266]
Lipopolysaccharides	Gram-negative bacteria	Indicator of pathogen infection	[266]

## Abbreviations

ALT: Alanine aminotransferase; Apo: Apolipoprotein; AsrA: Anaerobic sulfite reductase subunit A; AST: Aspartate aminotransferase; 5-AVAB: 5-aminovaleric acid betaine; BAs: Bile acids; BCAAs: Branched-chain amino acids; CA: Cholic acid; CAT: Cysteine aminotransferase; CBS: Cystathione β-synthase; CDCA: Chenodeoxycholic acid; COX: Cyclooxygenase; CRP: C-reactive protein; CTH: Cystathione γ-lyase; CVDs: Cardiovascular diseases; CysSSH: Cysteine persulfide; DCA: Deoxycholic acid; DHA: Docosahexaenoic acid; DMB: 3,3-dimethyl-1-butanol; EPA: Eicosapentaenoic acid; FDF: Fibroblast growth factor; FFAR: Free fatty acid receptor; FMO3: Flavin-containing monooxygenase 3; FOS: Fructo-oligosaccharides; FXR: Farnesoid X receptor; GCA: Glycocholic acid; GCDCA: Glycochenodeoxycholic acid; GLP-1: Glucagon-like peptide-1; GOS: Galacto-oligosaccharides; GPR43: G-protein-coupled receptor 43; GSSH: Glutathione persulfide; GWAS: Genome-wide association study; HDL: High-density lipoprotein; HMO: Human milk oligosaccharides; hsCRP: High-sensitivity C-reactive protein; ISAPP: International Scientific Association for Probiotics and Prebiotics; L-AAD: L-amino acid deaminase; LAB: Lactic acid bacteria; LCA: Lithocholic acid; LDL-C: Low-density lipoprotein cholesterol; LOX: Lipid oxygenase pathways; MPST: 3-mercaptopyruvate sulfurtransferase; NASH: Non-alcoholic steatohepatitis; NPC1: Niemann–Pick disease, type C1; oxLDL: Oxidized LDL; PAG: Phenylacetylglutamine; PBMCs: Peripheral blood mononuclear cells; PPDC: Phenylpyruvate decarboxylase; PPFOR: Phenylpyruvate–ferredoxin oxidoreductase; PSCR1: Proline/serine-rich coiled-coil protein 1; PUFAs: Polyunsaturated fatty acids; PYY: Peptide YY; RONS: Reactive oxygen or nitrogen species; ROS: Reactive oxygen species; RSnR: Polysulfides; RSSH: Hydropersulfide; SCFAs: Short-chain fatty acids; sICAM-1: Soluble intercellular adhesion molecule-1; SRB: Sulfate-reducing bacteria; TCA: Taurocholic acid; TCDCA: Taurochenodeoxycholic acid; TGR5: Takeda G-protein-coupled BA receptor; TMA: Trimethylamine; TMAO: Trimethylamine n-oxide; TMAVA: N,N,N-trimethyl-5-aminovaleric acid; TUDCA: Tauroursodeoxycholic acid; UDCA: Ursodeoxycholic acid; γBB: γ-butyrobetaine.
